# Scalable and Versatile Fabrication of Free-Standing Covalent Organic Framework Membranes with Tunable Microstructure for Molecular Separation

**DOI:** 10.1021/jacs.5c08788

**Published:** 2025-07-30

**Authors:** Kasper Eliasson, Fanfan Jiang, Michelle Åhlén, Maria Strømme, Chao Xu

**Affiliations:** † Division of Nanotechnology and Functional Materials, Department of Materials Science and Engineering, Ångström Laboratory, 8097 Uppsala University, SE-752 37 Uppsala, Sweden; ‡ State Key Laboratory of Materials-Oriented Chemical Engineering, College of Chemical Engineering, 478920 Nanjing Tech University, 211800 Nanjing, China

## Abstract

Covalent organic framework (COF) membranes hold significant promise for applications in separation, catalysis, and energy conversion; however, their industrial adoption has been hindered by the lack of scalable and efficient fabrication methods. Here, we present a fast, versatile, and broadly applicable strategy for fabricating free-standing and flexible COF membranes by casting precursor suspensions, followed by heat treatment under controlled humidity. This approach enables the fabrication of COF membranes with lateral dimensions up to several square decimeters and thicknesses that are tunable down to submicron levels within 1 h. It demonstrates remarkable versatility for producing a family of ketoenamine-linked COF membranes through the condensation of 1,3,5-triformylphloroglucinol with various amine monomers differing in length, side groups, and geometry. The resulting crack-free COF membranes exhibit high mechanical strength, with ultimate tensile strength up to 60 MPa and Young’s modulus up to 1.7 GPa, as well as exceptionally high porosity, with Brunauer–Emmett–Teller (BET) surface areas reaching up to 2226 m^2^ g^–1^. More importantly, the morphology, porosity, and crystallinity of the membranes can be finely tuned by modulating the heating conditions. The membranes with optimized microstructures demonstrate excellent separation performance, achieving over 99% rejection in nanofiltration of aqueous dye solutions, and a separation factor of 11 with an H_2_ permeance of 2857 GPU in H_2_/CO_2_ gas separation. This approach provides a scalable and effective pathway toward large-scale COF membrane manufacturing for advanced molecular separations and other membrane-based technologies.

## Graphical abstract

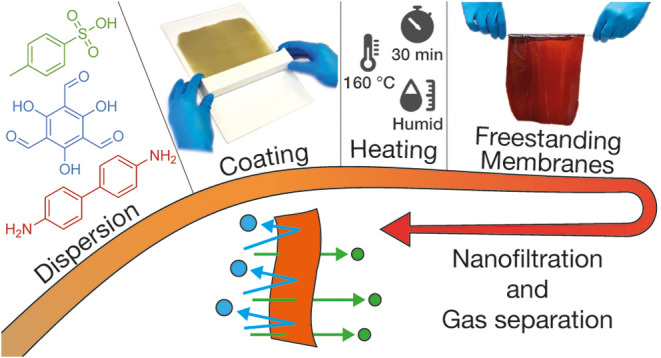

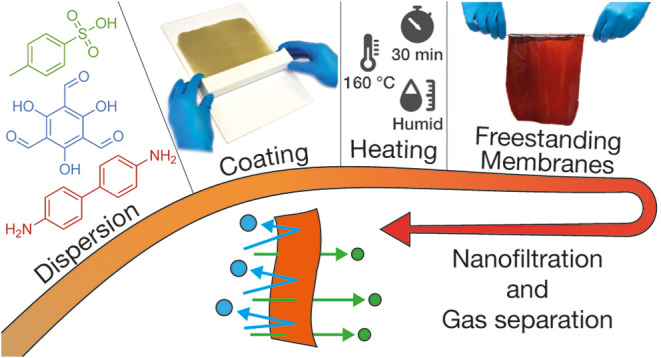

## 1 Introduction

Covalent organic frameworks (COFs) are a family of crystalline organic materials characterized by their highly ordered and porous structure. The materials are formed from the chemical interlinking of organic monomers via covalent bonds that lead to the formation of extended 2D or 3D network structures. The modular nature of COFs enables their pore size and functionalities to be tuned in a straightforward manner through the selection of organic monomers used in their construction.^1,2^ Framework structures with diverse linkages have therefore been assembled, among which imine- and ß-ketoenamine-linked COFs remain the most well-studied. In particular, COFs with ß-ketoenamine linking chemistry are noted for their exceptional thermal and chemical stability.^3^ These properties, in conjunction with their evenly sized nanopores, large pore volume, and modularity, make them highly promising materials for numerous applications such as catalysis, gas storage, energy storage, molecular separation, ion transport, and chemical sensing.^4,5^

COFs are typically synthesized by solvothermal methods as fine powders that require structuring to be usable in industrial settings. Traditional polymer processing techniques, such as molding and extrusion,^6^ are unsuitable for shaping these materials due to their insoluble and infusible nature. While certain COF powders can be compacted into relatively cohesive granules or sheets, this typically results in fragile structures. The mechanical strength can be improved by incorporating binders, although this introduces inactive materials that increase the dead volume and mass of the composite structures. The poor processability of COFs can be circumvented by direct synthesis of structured COFs by, for instance, sol–gel processes and growth on substrates or at interfaces.^7,8^ The synthesis of COF thin films, with submicron to a few micron thickness, is an area of especially high interest. Thin films exhibit high area-to-volume ratios, which facilitate rapid mass transport throughout the material, a critical parameter influencing performance in applications such as energy storage, molecular separation, catalysis, and chemical sensing.^5,9,10^ Moreover, the mechanical flexibility of thin films allows them to be readily folded, facilitating integration in compact devices. Additionally, the minimal material requirement per unit area can enhance their cost-effectiveness, particularly in membrane-based applications.^11^

Synthesizing COF films onto a macro-porous supporting layer is a widespread strategy for producing robust COF-composite membranes. For example, Pan et al.^12^ fabricated TpPa membranes — utilizing 1,3,5-triformylphloroglucinol (Tp) and p-phenylenediamine (Pa) monomers — on polyacrylonitrile substrates by infiltrating them with a water solution of Pa and acid, followed by a coating of Tp solution which reacted to form a ∼100 nm thick COF layer. In general, a supporting layer enhances the membrane’s mechanical strength, which is desirable for pressure-driven separation applications, but it also introduces inactive material, limits access to the support side of the membrane, and can reduce chemical compatibility and mechanical flexibility. Free-standing films circumvent these limitations, making them more suitable for applications that do not require high mechanical strength, involve harsh chemical environments, or demand unobstructed access to both sides of the film.

Several methods for the direct synthesis of free-standing ketoenamine-linked COF films have been reported, the majority of which can be categorized as either interfacial polymerization or casting methods.^13,14^ Liquid–liquid interfacial polymerization is the most common interfacial polymerization strategy for the preparation of free-standing films.^15,16^ The strategy is versatile and enables the fabrication of thin, defect-free films. However, in the context of large-scale production, it has significant limitations, including long synthesis times, high solvent demand, difficulty in maintaining large uniform liquid–liquid interfaces, and recovery of brittle free-standing films. Banerjee’s group introduced the casting strategy^17,18^ for the production of free-standing ketoenamine-linked COF films by adapting a powder synthesis method they had previously developed.^19^ The method, referred to as the “baking method”, involved mixing of organic monomers, *p*-toluenesulfonic acid (PTSA), and water into a paste that was cast into a film and subsequently heated. The PTSA molecules were found to serve a dual purpose by first interacting with the amine monomers and forming preorganized PTSA-amine salt fibers from which the COF structure could form, and second, acting as a hydrated acid catalyst that could regulate the Schiff base reaction. Furthermore, the method exhibits a high degree of versatility, demonstrated through the fabrication of free-standing films from several COFs. However, the reported films were thick — ranging from 25 to 700 μm — and displayed poor mechanical strengths. By using a similar strategy, thinner COF films with thicknesses down to 600 nm were prepared.^20,21^ The procedure involved dissolving monomers in a mixture of *N*-methyl-2-pyrrolidone (NMP) and dimethyl sulfoxide (DMSO), followed by casting and heating. Free-standing films of a sulfonated COF were gradually formed from the self-catalyzed reaction between the monomers, which exhibited good mechanical strength. However, versatility was not demonstrated by the method, as TpPa-(SO_3_) — composed of Tp and 2,5-diaminobenzenesulfonic acid (Pa-(SO_3_)) — was the only COF reportedly produced. Although this method can potentially be scaled up to produce large films, the reported procedures involved a lengthy heating process of 2–6 days, which is impractical for efficient large-scale production. Free-standing films can also be fabricated using alternative strategies such as layer-by-layer assembly of nanosheets^22^ and solution evaporation,^23^ which rely on COF growth through either diffusion-limited mixing of the organic monomers or through a PTSA-mediated strategy. However, previously reported methods generally lack the combination of versatility, scalability, and the ability to produce high-quality films with controlled microstructure and tunable thickness in the submicron to few-micron range.

Herein, we report a rapid, versatile, and scalable strategy for fabricating free-standing, mechanically robust yet flexible ketoenamine-linked COF films, with tunable thicknesses down to the submicron level and lateral dimensions up to 24 × 18 cm. The synthesis requires only 30 min of heating — significantly shorter than conventional methods — and yields films with exceptionally high surface area (up to 2226 m^2^ g^–1^) and excellent mechanical strength (Young’s modulus up to 1.7 GPa). By adjusting the humidity of the reaction environment to modulate the reversibility of polycondensation, we achieved precise control over the morphology, porosity, and crystallinity of the COF films. The COF films, with optimized microstructures, were applied as separation membranes and demonstrated excellent nanofiltration performance for removing organic dyes from wastewater, as well as outstanding H_2_/CO_2_ separation efficiency, exhibiting both high permeance and selectivity.

## 2 Results and Discussion

### 2.1 Synthesis of TpBd-COF Films

The synthesis of the free-standing COF films involves casting of the precursor suspension followed by heating under controlled humidity (Figure 1A). Briefly, PTSA and an amine monomer were mixed in a polar organic solvent at a 3:1 ratio (PTSA to NH_2_ group) to form a suspension of PTSA-amine salt particles. Tp was then added to the suspension, and the mixture was stirred overnight, after which it was doctor blade coated onto a glass plate and the solvent was allowed to evaporate. A glass lid was placed over the precursor film with a piece of wet glass wool absorbent attached to its ceiling. The purpose of the absorbent was to gradually release water vapor as the film was heated at 160 °C for 30 min. Partial melting of the precursors was visually indicated by the film’s appearance transitioning from matte to glossy, accompanied by a color change — typically from beige to deep orange or red — due to the occurrence of the Schiff base condensation. After cooling, the film was detached from the glass plate by submerging it in ethanol. A detailed description of the fabrication procedure and videos showing the coating and detaching steps are available in the Supporting Information. A model amine monomer, 4,4′-biphenyldiamine (Bd), was selected to study the synthesis process of the COF films due to its linear geometry and simple chemical structure.

**Figure 1:**
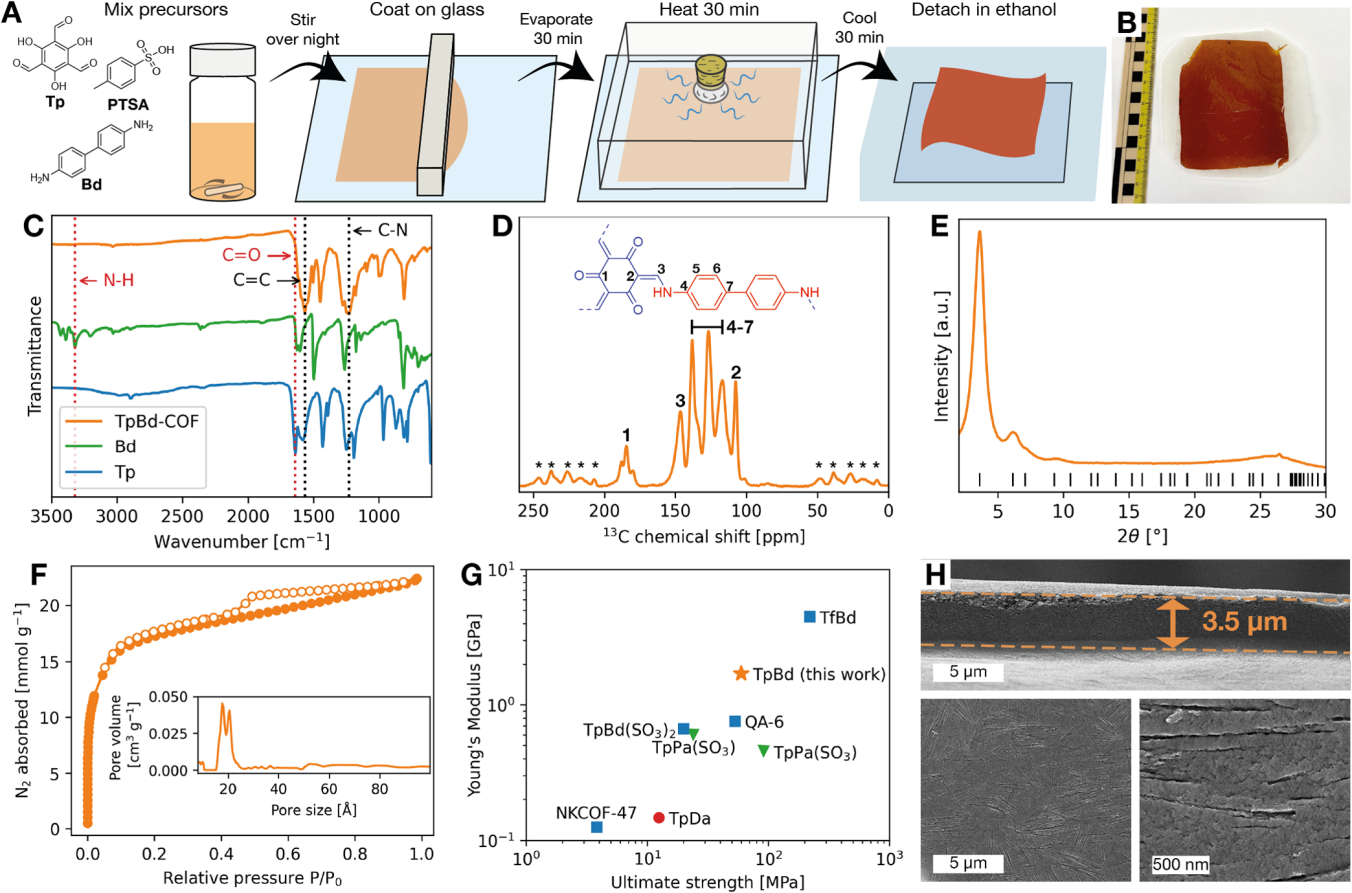
Fabrication procedure and characterization of TpBd-COF film: (A) Schematic fabrication procedure; (B) Optical image of the COF film; (C) FTIR spectra of the organic monomers and COF; (D) ^13^C MAS NMR spectrum of the COF; (E) PXRD diffractogram; (F) N_2_ sorption isotherm of the COF film recorded at – 196 °C. The inset shows its pore size distribution; (G) Mechanical properties determined by tensile testing compared to other reported free-standing COF membranes (symbols to denote fabrication method: ★ - this work, • - baking method,^18^ ■ - liquid-interfacial,^24–27^ ▼ - nanosheet assembly^22,28^ ); (H) SEM images of the cross section and top-down view of the COF film at low and high magnifications.

Notably, this method requires only 30 min of heating, which is significantly shorter than conventional approaches that typically take 8–72 h at a lower temperature of 60–120 °C.^17,18,21^ This represents a substantial improvement in processing efficiency and synthesis time. The dramatic acceleration in COF formation at elevated temperatures can be attributed to a combination of enhanced reaction kinetics and favorable phase behavior of the reactants. At higher temperatures, the condensation reaction between amine and aldehyde monomers proceeds more rapidly due to increased thermal energy, while the dynamic covalent bond exchange, which is critical for error correction and the crystallization process, is also more efficient. These factors enable the formation of porous and crystalline COF structures in a much shorter time.^29^ Additionally, the PTSA–organic monomer precursor, or the excess amount of PTSA, forms a molten phase at 160 °C. This melt-mediated environment promotes better mixing, enhances monomer mobility, and ensures uniform heat distribution, all of which further accelerate reaction kinetics and crystallization.^19^

An initial screening of several polar solvents — acetonitrile (ACN), dichloromethane (DCM), 2-propanol (IPA), tetrahydrofuran (THF), and water — was carried out to identify a suitable medium for dispersing the TpBd-COF precursors. All solvents yielded free-standing, crystalline TpBd films (Figures S1 and S2) of varying density and structural uniformity. Water produced films with a void-rich microstructure, while polar aprotic solvents, such as ACN, THF, and DCM, resulted in dense homogeneous films (Figure S3). Furthermore, incomplete dispersion of Bd, which was observed in the IPA solvent, gave rise to a film with an uneven surface, featuring bumps tens of microns in size (Figure S4). These results indicate that the selection of the solvent influences the solubility of the organic monomers and the dispersibility of the precursors, which can potentially affect both the homogeneity of the precursor film and the morphology of the resulting COF film. ACN was selected as the solvent for the following experiments as it produced a homogeneous film morphology and its low evaporation rate (compared to DCM and THF) simplified the coating procedure.

The molecular structure of the synthesized TpBd-COF film was characterized by Fourier transform infrared spectroscopy (FTIR) and solid-state ^13^C magic angle spinning (MAS) nuclear magnetic resonance (NMR) spectroscopy. The FTIR spectrum of the COF (Figure 1C) exhibited characteristic absorption bands at 1567 and 1230 cm^–1^, corresponding to the sp^2^ carbon–carbon (C=C) and amine (C–N) bonds of the ß-ketoenamine linkages, respectively. Furthermore, the absence of bands attributed to the carbonyl (C=O) and amine (N–H) bonds of the unreacted organic monomers at 1639 cm^–1^ and ∼3300 cm^–1^ suggests that they are fully consumed during the condensation and that the reaction proceeds to completion.^30^ Similarly, the ^13^C NMR spectrum (Figure 1D) of the COF displays a characteristic peak at ∼180 ppm corresponding to the carbonyl carbon (C=O) of the keto-form in the COF. The peaks at 150 and 105 ppm are assigned to enamine carbons (C=C–N), while the remaining signals in the 115–140 ppm range correspond to aromatic carbons. The lack of a peak at ∼ 190 ppm for the carbonyl carbon of the free aldehyde in the unreacted Tp,^31^ furthermore, lends support to the assumption that the polymerization reaction reaches completion and is consistent with the analysis of the IR spectrum of the COF. The FTIR and solid-state ^13^C NMR spectra are both in agreement with those previously reported for TpBd-COF.^17^ Analysis of the crystal structure of the COF was carried out by powder X-ray diffraction (PXRD). The diffractogram (Figure 1E) displays an intense peak at 3.6° and minor peaks at 6.1 and 26°, corresponding to the (100), (110), and (001) reflection planes, respectively. Pawley refinement (Figure 1E, Table S1) furthermore confirmed that the COF crystallized in an eclipsed AA-stacking configuration, which is consistent with those previously reported for TpBd-COF.^17^ The porosity of the COF film was evaluated by nitrogen (N_2_) sorption measurement at −196 °C (Figure 1F) and revealed that the material exhibited a Brunauer–Emmett–Teller (BET) surface area of 1472 m^2^ g^–1^, far exceeding those previously reported for TpBD-COF films.^17^ Furthermore, the pore size distribution (Figure 1F), calculated from the N_2_ adsorption isotherm using a nonlocal density functional theory model, confirmed that the COF possessed a pore size centered at 19 Å, which is comparable to the crystallographic pore diameter of ∼23 Å. The mechanical properties of the film were evaluated by tensile testing and revealed that the film possessed a high mechanical strength, characterized by a Young’s modulus of 1.7 GPa and an ultimate strength of 60 MPa, which are among the highest reported values for free-standing COF membranes (Figure 1G). Although the 3.5 μm thick film exhibited needle-shaped surface indentations approximately 10–50 nm in diameter, the overall morphology of the COF film was revealed to be free of any significant defects, such as pinholes, cracks, and large voids (Figure 1H). The high mechanical strength of the film was therefore attributed to its highly homogeneous and dense microstructure.

The successful fabrication of highly porous, crystalline, and structurally uniform COF films can be attributed to the combined effect of multiple key factors. First, the presence of water in the reaction system enhances the reversibility of the condensation reaction, facilitating the formation of large, well-ordered COF sheets. Second, the elevated reaction temperature partially melts the precursor material, increasing the mobility of the polymer sheets. This increased mobility aids the stacking of the sheets and promotes the formation of an ordered, porous network structure. Lastly, the thin film structure reduces temperature and water concentration gradients within the material, ensuring homogeneous conditions throughout the film.

### 2.2 Versatile Synthesis of COF Films

The generality of the synthesis method was evaluated through fabrication of eight different free-standing COF films using the following monomers: Pa, Bd, p-terphenylenediamine (Td), 4,4’-azodianiline (Azo), 3,3′-dinitrobenzidine (Bd­(NO_2_)_2_), 2,6-diaminoanthraquinone (Aq), 2,5-diaminobenzenesulfonic acid (Pa­(SO_3_)), and 4,4’,4”-(1,3,5-triazine-2,4,6-triyl)­trianiline (Tta) (Figure 2). Pawley refinement of the PXRD patterns of these COFs (Figure 2A–H, Table S1) was carried out to confirm their crystal structure. Intense peaks corresponding to the (100) plane were observed to be present in TpPa (4.8°), TpBd (3.6°), TpTd (2.9°), TpAzo (3.2°), TpBd­(NO_2_)_2_ (3.6°), and TpAq (3.5°).^19^ Peaks assigned to the (100) plane were also present in TpPa­(SO_3_) (4.9°) and TpTta (6.0°), albeit at a lower intensity, which indicates that these specific frameworks exhibit a higher degree of structural disorder compared to the other COFs. In the case of TpPa­(SO_3_), the lower crystallinity may be due to the bulky and ionic SO_3_H groups, which disrupts the formation of an ordered interlayer stacking by electrostatic repulsion and steric hindrance.^32^ The BET surface areas of the films were observed to be exceptionally high, ranging from 498 to 2226 m^2^ g^–1^ (Figure 2K), all exceeding those previously reported for free-standing films of the respective COFs (Table S2). Notably, TpPa, TpTd, TpBd­(NO_2_)_2_, TpAq, and TpPa­(SO_3_) even exceeded the highest, to our knowledge, reported BET surface areas for COF powders.^3,19,33,34^ Furthermore, the ability to produce highly crystalline and porous, free-standing COF films from organic monomers of varied geometries, functional groups, and from both ditopic and tritopic amine monomeric compounds demonstrates the remarkable versatility of this fabrication strategy.

**Figure 2:**
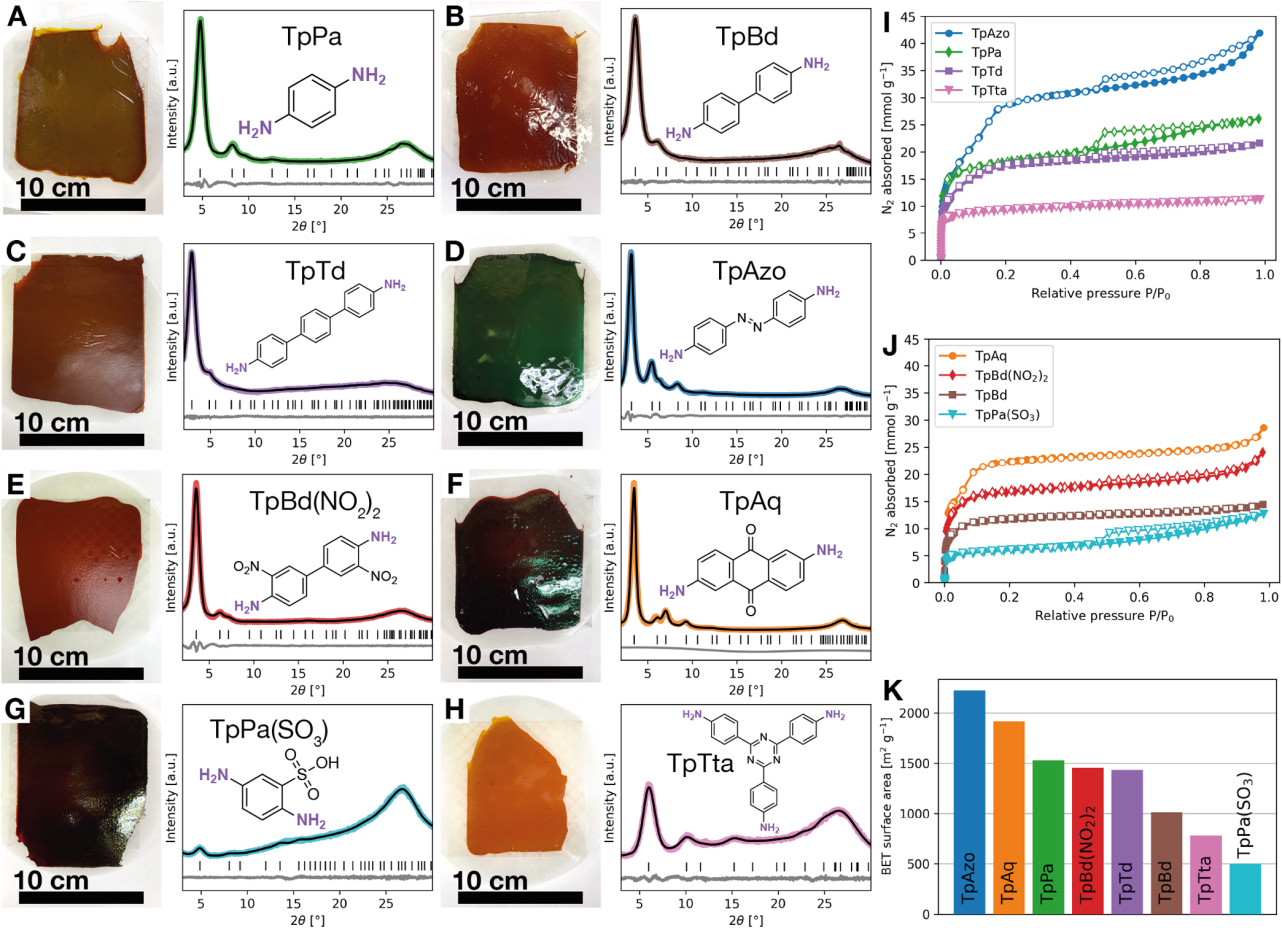
(A–H) Photos of free-standing COF films accompanied by their amine monomer’s structure and PXRD diffractogram. Colored line - experimental, black line - calculated, gray line - difference, black bars - Bragg positions (I, J) N_2_ sorption isotherms of the COF films recorded at −196 °C. (K) Bar chart of their BET surface areas.

### 2.3 Lateral Size and Thickness Modulation

Most reported free-standing COF films have been limited to lateral dimensions of only a few centimeters, significantly restricting their suitability for practical applications that demand larger-area films. In contrast, our standard synthesis procedure enables the fabrication of substantially larger free-standing COF films, up to 10 × 10 cm in size. To evaluate the scalability of this method — crucial for practical and industrial applications — we conducted a scaled-up synthesis of a TpBd film (Figure 3A). This was performed by following the same fabrication protocol while proportionally increasing the amounts of starting materials and solvents, and employing a larger doctor blade and glass substrate. Remarkably, the scaled-up synthesis produced a large, crack-free, and flexible free-standing TpBd film with dimensions of approximately 24 × 18 cm. To the best of our knowledge, this is the largest freestanding COF film reported to date. PXRD analysis confirmed that the crystalline structure of the large film was preserved and comparable to that of the standard 10 × 10 cm film (Figure 3B). Furthermore, both films exhibited nearly identical N_2_ adsorption behavior at relative pressures below 0.1 (Figure 3C), with BET surface areas of 1110 and 1014 m^2^ g^–1^ for the smaller and larger films, respectively. The thickness of the TpBd films was estimated from their SEM cross sections (Figure 3D,J), with the larger and smaller TpBd films approximately 21 and 18 μm thick, respectively. Furthermore, both films exhibited homogeneous morphologies aside from some minor voids that were ∼1–3 μm in size. No significant structural differences between the films were found, other than what can be attributed to batch-to-batch variability – thus confirming the robustness of the fabrication strategy and its capability for producing COF films of varying lateral dimensions with reproducible crystallinity, porosity, and morphology. Furthermore, PXRD and SEM analysis of a large 24 × 18 cm TpBd-COF film (Figure S5) demonstrated a high uniformity in both crystallinity and surface morphology between different regions of the film as well as a low thickness variation, which varied between 12.5 and 18.5 μm. It should be noted that the dimensions of the scaled-up COF film were constrained by the size limitations of the laboratory equipment (e.g., glass substrates, ovens, doctor blades, and lids); however, these limitations can be easily addressed in industrial-scale production aiming to fabricate even larger films.

**Figure 3:**
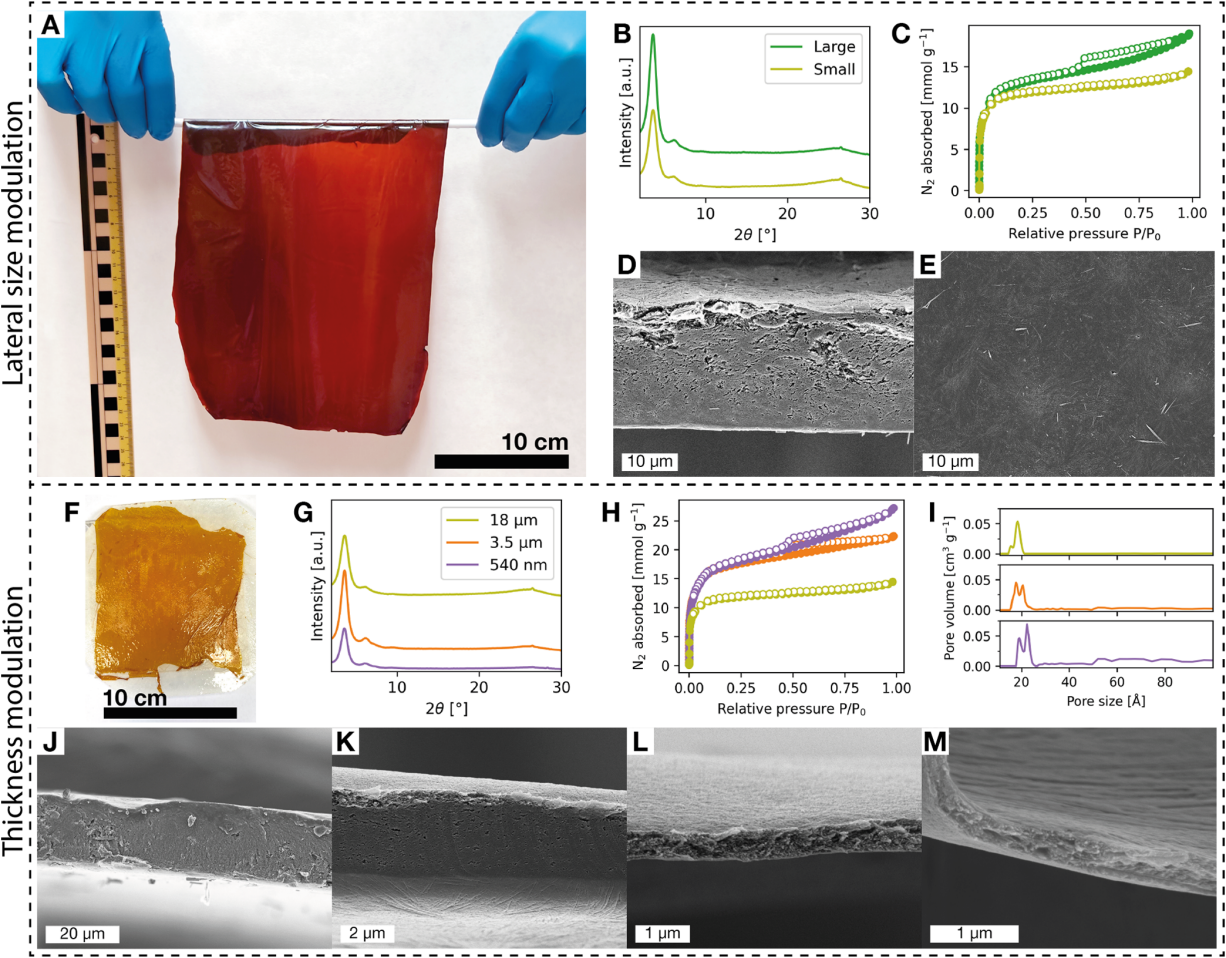
(A) Optical image of large TpBd-COF film. (B) PXRD patterns of the large and small COF films. (C) N_2_ sorption isotherms of the large and small films recorded at −196 °C. (D, E) SEM images of large film. (F) Optical image of the 540 nm-thick film. (G) PXRD diffractograms of the COF films with varied thickness. (H) N_2_ sorption isotherms of COF films with varied thickness recorded at −196 °C and (I) their pore size distributions. (J–M) SEM cross-section images of films with thicknesses of: 18 μm, 3.5 μm, 540 nm, and 370 nm. The film labeled “Small” and “18 μm” is the same TpBd film as presented in Figure 2. The film labeled “3.5 μm” is presented in Figure 1.

Membrane thickness is a critical parameter governing performance in liquid nanofiltration, gas separation, and ion transport.^10^ The trade-off relationship between mechanical robustness and mass transport resistance necessitates precise thickness control to meet application-specific requirements. As such, the thickness of the TpBd-COF films was modulated by two methods. First, by varying the doctor blade gap width, and second, by tuning the concentration of the precursor suspension. The standard fabrication procedure yielded a 3.5 μm thick film (previously presented in Figure 1). By increasing the doctor blade gap from ∼50 to ∼250 μm, an 18 μm-thick film was produced. The change in thickness was observed to be proportional to the change in blade gap. By diluting the precursor suspension with additional ACN to 33 and 20% of the standard concentration, significantly thinner films with thicknesses of 540 and 370 nm were fabricated. The thickness reduction was disproportionately large relative to the change in precursor concentration, most likely due to the dilution lowering the suspension’s viscosity and contributing to a thinner coating. The reduced thickness of the 370 nm film gave rise to poor mechanical stability, resulting in the film breaking into small fragments when it was detached from the glass substrate. However, the other films could be detached intact, including the 540 nm-thick film (Figure 3F). The SEM cross sections (Figure 3J–M) reveal highly homogeneous morphologies in the 3.5 μm, 540 nm, and 370 nm-thick films, while the 18 μm-thick film contained minor voids, approximately 1–3 μm in size. The 18 μm, 3.5 μm, and 540 nm-thick films were further evaluated by PXRD and N_2_ sorption. All films displayed similar diffraction patterns and only showed a discrepancy in the overall intensity of the diffraction peaks (Figure 3G). It was noted that the pulverized 540 nm-thick sample packed more loosely than the thicker samples when they were prepared for PXRD. This may explain its lower PXRD intensity, as all of the samples were loaded into holders of identical volume. The N_2_ sorption isotherms of the COFs displayed lower uptake for the 18 μm-thick film, as compared to the 3.5 μm and 540 nm-thick films, which had nearly identical N_2_ uptake, particularly at relative pressures below 0.1. The BET surface areas were calculated to be 1014, 1472, and 1523 m^2^ g^–1^ for the 18 μm, 3.5 μm, and 540 nm-thick films, respectively. Overall, films of submicron thickness may be produced by dilution of the precursor suspension without compromising their porosity or morphology. Thicker films can be produced by increasing the doctor blade gap, although in our case, this resulted in a slightly reduced porosity and less homogeneous morphology. The suboptimal structure of the thick film may be attributed to concentration gradients created as the solvent evaporates, which would be more pronounced in thicker films.

### 2.4 Modulation of Microstructure

Since water plays an essential role in COF synthesis through the reversible Schiff base condensation reaction, we hypothesized that controlling the humidity in the environment could influence the microstructure, such as crystallinity, porosity, and morphology, of the resulting COF films.^19^ During the method development, we observed that heating the film in a closed humid system increased the crystallinity of the product while, at the same time, significantly affecting the morphology of the resulting film. The influence of the humidity during heating was therefore investigated in more detail at four different conditions (C1–C4) (Figure 4A): C1 - Open system; C2 - Closed system with absorbent wetted with 0.1 mL water; C3 - Closed system with absorbent wetted with 1 mL water (standard procedure); and C4 - Closed system with the inside of lid, wetted with 1 mL water. In C4, the inside of the glass lid had been sprayed with water droplets that evaporated rapidly as the lid temperature increased, whereas in C2 and C3, the water was added to absorbent glass wool suspended from the lid and evaporated gradually as the temperature of the enclosed air increased.

**Figure 4:**
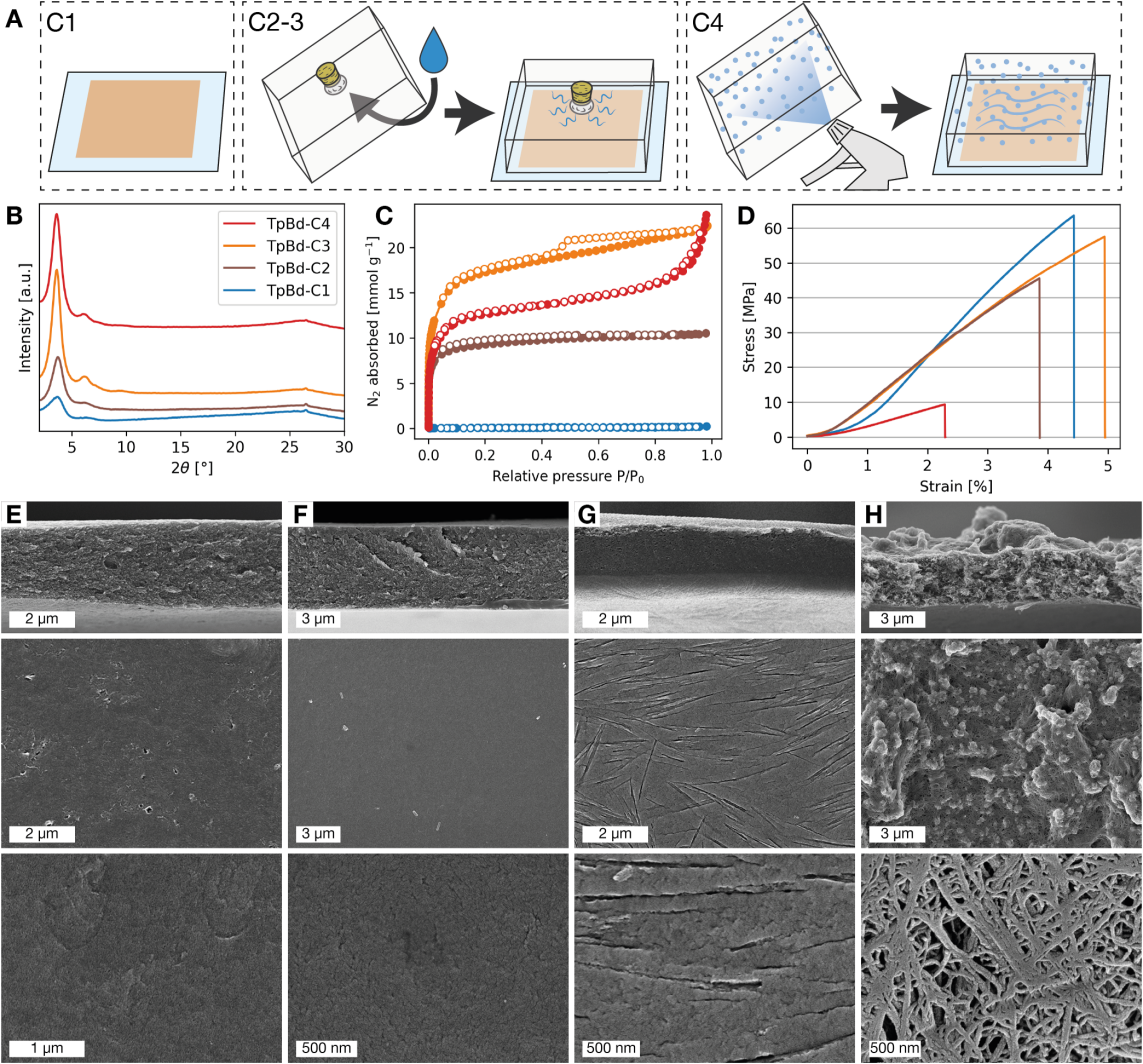
(A) Schematic representation of heating conditions: C1 - Heated open; C2–3 - Heated covered, absorbent wetted with 0.1 mL (C2) or 1 mL (C3) water; C4 Heated covered, lid sprayed with 1 mL water. (B) PXRD pattern of TpBd-C1 to C4. (C) N_2_ sorption isotherms of TpBd-C1 to -C4 recorded at – 196 °C. (D) Stress–strain curves of TpBd-C1 to -C4, (E–H) SEM cross-section and top view images at varying magnifications of: (E) TpBd-C1, (F) TpBd-C2, (G) TpBd-C3, (H) TpBd-C4. The film labeled “TpBd-C3” is presented in Figure 1 and in Figure 3 as “3.5 μm”.

The PXRD patterns of TpBd-C3 and TpBd-C4 (Figure 4B) display distinct peaks at 3.6 and 6.1°, consistent with those expected for TpBd COF. The low-humidity samples (i.e., TpBd-C1 and TpBd-C2) both exhibited similar patterns, although the films were observed to have an overall lower crystallinity. These observations agree well with the N_2_ sorption results (Figure 4C), films with lower crystallinity correlating to a reduced microporosity. Furthermore, TpBd-C4, -C3, and -C2 were found to have BET surface areas of 1081, 1472, and 797 m^2^ g^–1^, respectively, while TpBd-C1 showed negligible N_2_ uptake. In particular, the N_2_ isotherm for TpBd-C4 displayed a sharp increase in uptake above a relative pressure of 0.85, indicating the presence of large mesopores. The three lower humidity samples (i.e., TpBd-C1, -C2, and -C3) were observed to show decreasing porosity with lower humidity and were therefore additionally characterized by CO_2_ adsorption at 0 °C (Figure S6). The CO_2_ uptakes of TpBd-C2 and -C3 were both 2.5 mmol g^–1^ at 1 bar, while TpBd-C1 had an uptake of 1.5 mmol g^–1^. This confirms the presence of micropores in TpBd-C1 that may either be too small or inaccessible to be probed by N_2_ under cryogenic conditions. Tensile testing was performed to investigate the mechanical properties of the films. A representative stress–strain curve for each sample is presented in Figure 4D, and additional data are available in Table S3. The TpBd-C1, -C2, and -C3 films exhibited a mechanical strength and stiffness toward the upper end of the typical range for COF membranes (ultimate strengths of 4–200 MPa and Young’s moduli of 0.1–2.0 GPa,^24^ Table S4), with ultimate strengths of 63, 46, and 58 MPa and Young’s moduli of 1.4, 1.4, and 1.9 GPa, respectively. TpBd-C4, on the other hand, was significantly weaker and exhibited an ultimate stress of 9.0 MPa and Young’s modulus of 0.50 GPa. The low strength of TpBd-C4 can be attributed to its void-rich microstructure. SEM imaging (Figure 4E–H) showed that the TpBd-C1, -C2, and -C3 films possessed dense morphologies, while TpBd-C4 had a microstructure consisting of fibrous particles 30–100 nm in diameter and surface protrusions several microns large. Coherence scanning interferometry mapping (Figure S9) furthermore shows that the surface roughness of TpBd-C4 was comparable to that of the unheated precursor film, with mean height (**Sa**) values of 1.0 and 2.6 μm, respectively, while TpBd-C3 was significantly smoother with a **Sa** of 65 nm.

The morphological differences observed in the resulting COF films can be attributed to the critical role of humidity in regulating the formation and stabilization of fibrous PTSA–amine intermediates during synthesis. Water molecules contribute by (1) forming hydrogen bonds with both PTSA and amine groups, thereby maintaining the structural integrity of the supramolecular fibrous assemblies, and (2) enhancing molecular mobility, which facilitate the self-assembly of the components into ordered fibrous structures.^17,19,35^ Under high humidity, these stabilized fibers persist and serve as templates for COF growth into fibrous aggregate structures, as observed in TpBd-C4. In contrast, under drier conditions, where the fibrous PTSA–amine intermediates are less stable, smaller, low-aspect-ratio COF particles form, resulting in a denser film. The formation of larger COF fibers under highly humid conditions may also result from slower reaction kinetics, as water lowers the rate of the initial Schiff base condensation step, promoting directional growth and larger particle formation.

### 2.5 Nanofiltration and Gas Separation

Given their high mechanical strength and nanoporous, defect-free structure, we envision that the COF films could serve as promising membranes for various separation processes, including nanofiltration for solvent purification, removal of organic micropollutants in water treatment, and gas separation.^13^ First, the TpBd films were evaluated as nanofiltration membranes for rejection of Congo red dye molecules from aqueous solutions, and the effect of humidity during COF synthesis on their nanofiltration performance was investigated. Specifically, nanofiltration experiments were conducted using a home-built setup with a stirred cell and slow cross-flow (Figures S10 and S11). The filtration was performed on an aqueous Congo red solution with a concentration of 10 ppm. Both COF films, synthesized under dry conditions (TpBd-C1) and intermediate humidity conditions (TpBd-C2 and C3), exhibited high rejection rates of >99% (Figure 5A and Table S6). However, TpBd-C2 and TpBd-C3 exhibited higher permeance values of 15 and 30 L m^–2^ h^–1^ bar^–1^, respectively, compared to TpBd-C1 (4.3 L m^–2^ h^–1^ bar^–1^), which can be attributed to the increased porosity of TpBd-C2 and TpBd-C3 under higher humidity conditions. In contrast, TpBd-C4 synthesized at the highest humidity showed a slightly lower rejection rate of 90%, but a significantly higher permeance of 86 L m^–2^ h^–1^ bar^–1^. This enhanced permeance and slightly reduced rejection performance are likely due to the formation of macropores at high humidity. The dye rejection performance of the TpBd films is comparable to that of previously reported free-standing^17^ and substrate-bound^36^ TpBd-COF membranes, which exhibited rejection rates of 96 to 99.5% and water permeance ranging from 34 to 90 L m^–2^ h^–1^ bar^–1^. These results demonstrate the trade-off effect of porosity on nanofiltration performance: increasing membrane porosity enhances permeance but compromises the rejection rate. This suggests that carefully tuning the porosity is crucial for optimizing overall nanofiltration efficiency. Additionally, a long-term filtration test was carried out using the TpBD-C3 membrane to evaluate its operational stability (Figure 5B). After continuous filtration for over 16 h, the rejection rate remained consistently above 98.5%, while the water permeance decreased by approximately 40% from the initial value, likely due to pore blockage caused by dye accumulation. Notably, the TpBD-C3 membrane exhibited a low adsorption capacity of ∼21 mg g^–1^ for Congo red at a concentration of 10 ppm (Figure S12). This corresponds to a maximum adsorption of ∼0.02 mg of Congo red by the membrane piece used in the nanofiltration experiment, accounting for <1% of the total dye rejected during the 16-h filtration. These results clearly indicate that size exclusion, rather than adsorption, is the dominant mechanism responsible for the high dye rejection observed in the nanofiltration experiments.

**Figure 5:**
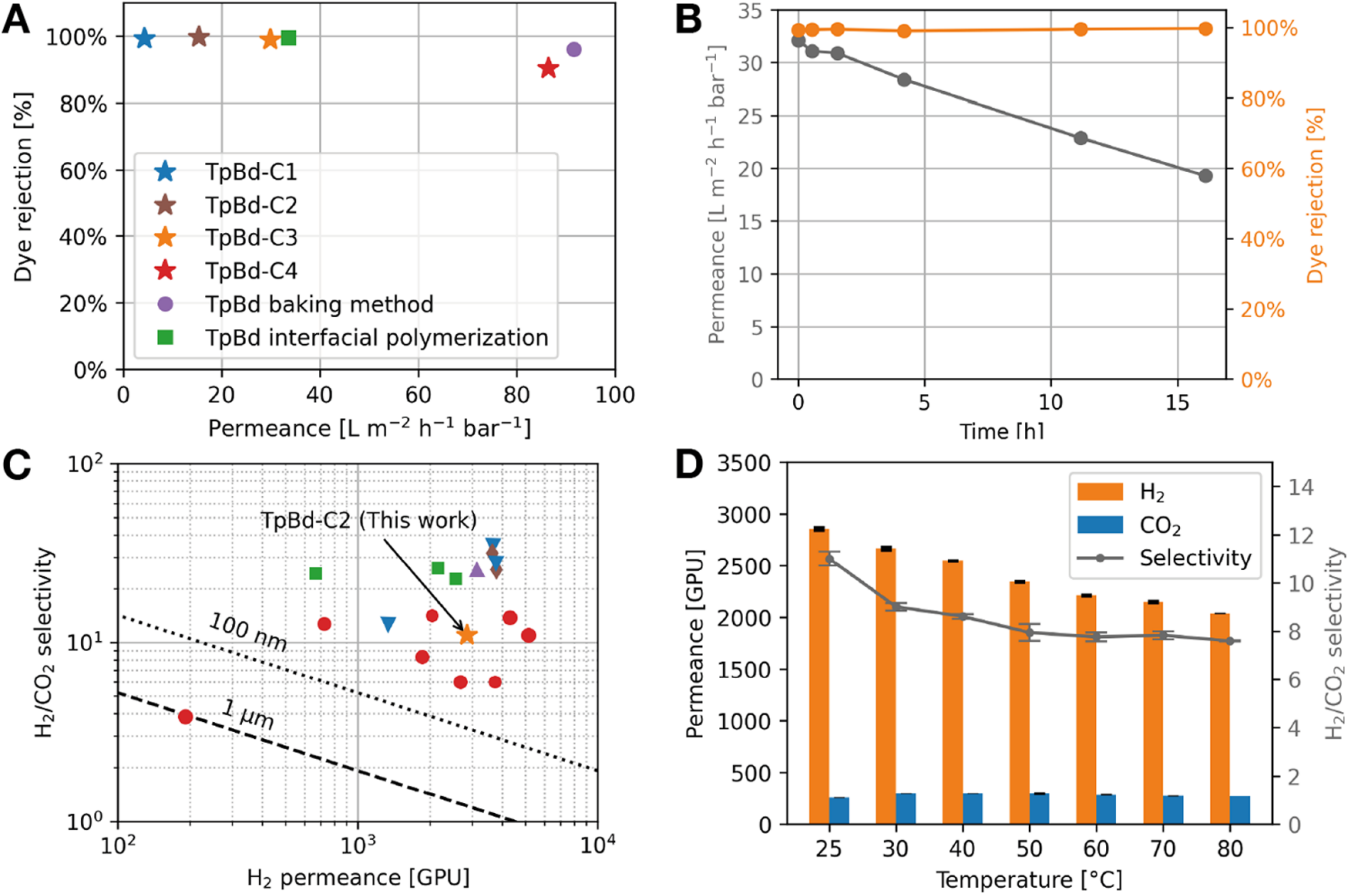
(A) Nanofiltration performance of Congo red aqueous solution using our TpBd membranes compared with reported membranes fabricated by the baking method^17^ and interfacial polymerization.^36^ (B) Results of extended Congo red filtration experiment. (C) Mixed gas H_2_/CO_2_ separation performance of TpBd-C2 compared to various reported membranes: ★ this work, • Pristine COF,^37–42^ ■ COF-COF composite;^42–44^ ▼ COF-MOF composite,^40,45^ ▲ COF-GO composite,^46^ ⧫ vertically aligned COF,^47^ Dashed and dotted lines - Robeson (2008) upper bound assuming 1 μm and 100 nm membrane thickness, respectively.^48^ (D) TpBd-C2 gas permeation result for H_2_/CO_2_ (1:1, v/v) at 0.2 MPa transmembrane pressure with varying temperature (permeability - bars, left axis; H_2_/CO_2_ selectivity - points, right axis).

It is worth highlighting that although TpBd-C3 and -C4 contained meso- and macropores, as revealed by N_2_ adsorption measurements and SEM images, respectively, which are significantly larger than the molecular size of Congo red, they still exhibited a relatively high rejection rate. This performance can be attributed to several synergistic effects. First, the dense packing of COF nanoparticles/nanofibers forms a tortuous transport pathway, enhancing size-exclusion through interparticle voids.^49^ Second, the large surface pores may act as adsorption sites for Congo red molecules, leading to a surface accumulation that alters the membrane’s surface charge. This adsorbed dye layer can impart an overall positive charge to the membrane, promoting electrostatic repulsion against further Congo red molecules in the solution. Third, the accumulation of dye molecules on the membrane surface could progressively clog the larger pores, as indicated by the decreased permeance observed for TpBd-C3 during the long-term continuous filtration experiment. This pore blockage effectively reduces the pore size and further enhances the rejection rate. These combined effects contributed to the overall high dye rejection performance of the TpBD-C4 membrane despite the presence of large pores.

Additionally, the TbBd membranes were evaluated for H_2_/CO_2_ separation, which is relevant for applications such as hydrogen purification, syngas upgrading, and carbon capture. Single-gas permeation tests revealed that the TbBd-C2 membrane, synthesized under low-humidity conditions, exhibited promising performance, with a high H_2_ permeance of 2340 GPU and an ideal H_2_/CO_2_ selectivity of 19 at 25 °C and a transmembrane pressure of 0.3 MPa. These values are comparable to the best-performing COF membranes reported to date. In contrast, other tested membranes displayed either significantly lower selectivity or very low permeance, likely due to excessively large pore sizes or overly dense structures. Hydrogen separation from syngas constitutes an important step in many industrial processes,^50^ and is typically carried out at elevated temperatures ranging from 100–300 °C.^51,52^ Therefore, to further assess the membrane’s practical potential, the TbBd-C2 membrane was subjected to binary gas permeation tests using an equimolar H_2_/CO_2_ mixture (1:1, v/v) at temperatures ranging from 25 to 80 °C, under a fixed transmembrane pressure of 0.2 MPa (Figure 5D, Table S8). The membrane achieved a relatively high separation factor of 11.0 at 25 °C, which gradually decreased to 7.6 as the temperature increased to 80 °C. This decline may be attributed to a competitive diffusion mechanism, where CO_2_ diffuses more easily through the membrane at elevated temperatures, thereby hindering the transport of H_2_ and reducing the overall H_2_/CO_2_ selectivity.^38^ Notably, the separation performance surpasses the 2008 Robeson upper bound even when a membrane thickness of 100 nm is considered. 100 nm is significantly thinner than our few micron thick membrane, but representative of the COF composite membranes included in the comparison, which generally utilize a submicron thick layer of active material (Figure 5C, Table S7).^53^

Since TpBd-COF has an intrinsic pore size of approximately 2.0 nm – substantially larger than the kinetic diameters of H_2_ (2.89 Å) and CO_2_ (3.3 Å), size exclusion in the main channels is unlikely to be the dominant separation mechanism. Moreover, the observed H_2_/CO_2_ selectivity is much higher than the ideal Knudsen selectivity of 4.7, which rules out Knudsen diffusion as the dominant mechanism. Although the dense microstructure of the TpBd-C2 membrane (Figure S13) may create tortuous diffusion pathways that could increase the residence time of molecules and potentially enhance the separation efficiency, the comparatively large dimensions of these pathways, however, do not provide the size exclusion mechanism typically associated with molecular sieving.^38^ The observed separation selectivity may instead be influenced by stronger CO_2_–framework interactions. Given that TpBd-C2 exhibited relatively high CO_2_ adsorption capacity (2.5 mmol g^–1^ at 1 bar and 0 °C), CO_2_ molecules are likely to experience stronger dipole–quadrupole interactions within the COF structure than H_2_.^54^ This enhanced adsorption could result in slower CO_2_ diffusion, which in turn contributes to the higher H_2_/CO_2_ selectivity by impeding the transport of CO_2_ while allowing H_2_ to permeate more easily.^38^

## 3 Conclusions

In summary, we have developed a rapid, scalable, and versatile strategy for fabricating a family of free-standing, crack-free, and mechanically strong COF membranes, demonstrating excellent performance in both aqueous nanofiltration and gas separation. This synthesis method reduces production time and solvent consumption through a straightforward process of precursor casting followed by controlled thermal treatment, significantly enhancing efficiency and sustainability compared to existing approaches. The simplicity, scalability, and adaptability of the method enable the fabrication of COF membranes and their composites with tailored structures and properties at a scale relevant for industrial deployment, advancing their practical applications in advanced membrane technologies.

It was observed that humidity during thermal treatment significantly influences the porosity, crystallinity, and morphology of the COF films. Specifically, low humidity conditions led to the formation of amorphous, low-porosity films, whereas high humidity resulted in highly crystalline films with void-rich macroporous structures. Intermediate humidity levels facilitated the formation of dense, microporous films. Further studies, such as in situ characterizations during synthesis, are needed to elucidate the fundamental mechanisms governing these humidity-dependent structural variations, which may guide the rational design of COF films with well-tailored architectures for specific targeted applications.

Despite the significant advantages of the developed COF membranes, several challenges must be addressed before they can be used in industrial applications. For instance, larger membrane sizes on the order of square meters are required for practical deployment. Additionally, costs need to be further reduced, such as by employing more cost-effective organic monomers. Moreover, the long-term aging stability and operational durability of COF membranes should be improved. Finally, integrating COF membranes into existing module designs will be essential for successful industrial implementation. Overcoming these challenges is critical to unlocking the full potential of COF membranes for scalable and efficient industrial separations.

## Supplementary Material







## Notes

Experimental section, additional results, figures, and tables (PDF)
Video of coating procedure (MP4)
Video of COF film being detached from glass substrate (MP4)
