# Supplementary material for: Scalable and Versatile Fabrication of Free-Standing Covalent Organic Framework Membranes with Tunable Microstructure for Molecular Separation
Source: J Am Chem Soc. 2025 Jul 30;147(32):29271–81. doi: 10.1021/jacs.5c08788 (PMC12356585; doi:10.1021/jacs.5c08788)
Supplement: Supplementary file 1 [file ja5c08788_si_001.pdf]

Supporting Information: Scalable and Versatile Fabrication of Free-standing Covalent Organic Framework Membranes with Tunable Microstructure for Molecular Separation

Kasper Eliasson,† Fanfan Jiang,‡ Michelle Åhlén,† Maria Strømme,† and Chao Xu*,†

†*Division of Nanotechnology and Functional Materials, Department of Materials Science and Engineering, Ångström Laboratory, Uppsala University, SE-752 37 Uppsala, Sweden.*

‡*State Key Laboratory of Materials-Oriented Chemical Engineering, College of Chemical Engineering, Nanjing Tech University, 211800, Nanjing, China*

E-mail: chao.xu@angstrom.uu.se

Contents

Materials and methods **S-3**

Materials S-3

Membrane fabrication S-3

Characterization S-5

Dye nanofiltration S-8

H_2/CO_2 separation S-9

Supplementary data **S-11**

Solvent screening S-11

Pawley refinement S-14

Reported BET surface areas S-15

Uniformity of large membrane S-17

CO_2 sorption isotherms S-18

Pore size distributions S-18

Tensile testing S-19

Coherence scanning interferometry maps S-19

Density and swelling rate S-19

Dye nanofiltration S-20

Reported H_2/CO_2 separation performances S-22

High-resolution TEM image S-24

Thermogravimetric analysis S-24

Materials and methods

Materials

p-phenylenediamine (Pa) was purchased from Fisher Scientific. 4,4'-biphenyldiamine (Bd), *p*-terphenylenediamine (Td), 4,4'-Azodianiline (Azo), 3,3'-Dinitrobenzidine ($Bd(NO_2)$), 2,6-Diaminoanthraquinone (Aq), 2,5-Diaminobenzenesulfonic acid ($Pa(SO_3)$), 4,4',4"-(1,3,5-Triazine-2,4,6-triyl)trianiline (Tta), and 1,3,5-triformylphloroglucinol (Tp) were purchased from Tensus Biotech. All monomers had a purity of >95%. *p*-toluene sulfonic acid monohydrate ($PTSA{\cdot}H_2O$) and solvents were purchased from VWR Sweden. All reagents were used without additional purification.

Membrane fabrication

The synthesis procedures for all of the samples are almost identical. The scale of the precursor suspension was varied, and in some cases, additional solvent was added to adjust the dispersion's viscosity. We start by presenting the general method, followed by any additional modification that were made.

General procedure

The amine monomer (2 mmol) was ground to a fine powder and added to a 20 mL vial with $PTSA{\cdot}H_2O$ (12 mmol) and 10 mL acetonitrile (ACN). The mixture was stirred for 30 minutes, after which Tp (1.33 mmol) was added. The mixture was stirred overnight and a portion of it was transferred onto a borosilicate glass sheet and evenly spread into a 10 x 10 cm film using a polytetrafluoreten (PTFE) doctor blade with a ∼50 µm gap. The solvent was evaporated from the film for 30 minutes at room temperature and 55% relative humidity. If the film was held at a lower relative humidity of 40%, we would observe the formation of a inhomogeneous reticulated pattern in the film. Uneven edges on the film were trimmed using a razor. A lid was thereafter prepared, by suspending a piece of glass wool from a

600 mL glass box, after which 1 mL water was added to the wool and the lid was placed over the film. The setup was placed in a convection oven set to 160 °C on top of spacers, to not directly contact the metal grating in order to ensure even heating from the bottom. After 30 minutes, the setup was removed from the oven and the lid removed. The plate was allowed to cool and then submerged in ethanol. After a few minutes, the film was detached by gently agitating the plate. Films were washed by placing them between polypropylene meshes, then stacked flat on top of each other in a large volume Soxhlet extraction setup. Washing in methanol proceeded for at least 12 hours, draining about once per hour. After washing, the membranes were placed between filter papers and stored submerged in ethanol. The membranes were dried in preparation for SEM and tensile testing. To accomplish this, they were placed between filter papers under gentle pressure in a container that restricted air flow and left for several days. Despite our best efforts to dry them gently, most of the membranes cracked to some degree during drying.

Modifications to the general procedure

TpBd-Large - Coated with a wider PTFE doctor blade with a ∼200 µm gap. A lid with a volume of 3100 mL was used with 5 mL water added to the absorbent.

TpBd precursors in different solvents - ACN was replaced with dichloromethane (DCM), 2-propanol (IPA), tetrahydrofuran (THF) or water for the respective experiments. Films coated with a ∼250 µm doctor blade gap

COFs other than TpBd - The amine monomer was replaced and films were coated with a 250 µm blade gap. **Additionally:**

- **TpPd** - 2-propanol was used in place of ACN as ACN produced a grainy suspension.
- **TpTta** - Amine monomer amount was adjusted to 1.33 mmol to maintain an even linking group ratio.

TpBd-18µm - Coated with a ∼250 µm doctor blade gap.

TpBd-540nm and TpBd-370nm - The precursor suspensions were diluted to 33% or 20% of their standard concentration by adding additional ACN and mixing for 1 hour.

TpBd-C1 - Heated without a lid.

TpBd-C2 - Heated under a lid with 0.1 mL water added to glass wool absorbent suspended from the lid.

TpBd-C4 - Heated under a lid, the inside of which had been sprayed with 1 mL water.

Characterization

Powder X-ray diffraction (PXRD)

Membranes were ground to a powder and oven dried at 70°C. Analysis was performed on a Bruker D8 Advance diffractometer equipped with a motorized air-scatter slit, using Cu Kα radiation and a Bragg-Brentano detector geometry.

Scanning electron microscopy (SEM)

Dried membranes were cut into thin strips that were attached on SEM-stubs using carbon tape for top face imaging, or to the side of the stubs cross sections facing up using kapton tape. The samples were coated with a thin layer of Au/Pd prior to imaging. Imaging was performed on either a Zeiss Leo 1530 or Zeiss Leo 1550 using an acceleration voltage of 5 keV and an in-lens or Everhart-Thornley detector.

High-resolution transmission electron microscopy (HR-TEM)

HR-TEM images were recorded on a JEOL JEM-F200 microscope equipped with a Gatan Rio camera.

Gas sorption measurements

Membranes were ground to powders and oven dried at 70 °C. The samples were then loaded into glass tubes and degassed for 6 hours at 120 °C. N_2 sorption was performed at -196 °C on a Micromeritics Tristar II plus. However, for TpBd-C1 to -C3, a Micromeritics ASAP 2020 was used for the 0 °C CO_2 and -196 °C N_2 sorption experiments. The MicroActive software (version 5.01) was used for data treatment. BET surface areas were calculated from N_2 adsorption isotherms using the "bet_autofit_v0712" python script included in the software. We note that the BET fitting script validates that the BET linear fit has correlation coefficient > 0.995, parameter $C > 0$, and that the Rouquerol criteria are satisfied. Langmuir surface areas were calculated from CO_2 adsorption isotherms in the 7-40 kPa pressure interval. DFT-pore size distributions were calculated from the N_2 adsorption isotherms using the "N2 - Cylindrical Pores - Oxide Surface" model and a regularization of 0.01. The Horvath-Kawazoe (HK) pore size distributions were calculated from N_2 adsorption isotherms using a "Cylinder (Saito-Foley)" geometry, an interaction parameter of 3.490 e^{-43} erg cm^4, and Cheng-Yang correction applied.

Coherence scanning interferometry

COF films were dried and attached to glass slides with double-sided tape. The precursor film was coated on a glass slide and dried at 70 °C. Characterization was performed on a Zygo Nexview instrument, using a 50x Mirau objective. Surface maps were flattened by 4th-degree polynomial background subtraction.

Tensile testing

Dried membranes were cut into 12 x 30 mm strips using a razor. The ends of the strips were covered with painter's tape. Sample dimensions were measured with a ruler. The thickness was taken as the mean of 4 points per sample measured with a micrometer gauge. The samples were mounted by clamping them along the edges of the tape. The analysis

was performed on a Shimadzu Autograph AGS-X. Figure S8 shows examples of the tensile testing samples.

Fourier transform infrared spectroscopy (FTIR)

Pulverized membranes were measured by attenuated total reflection Fourier transform infrared (ATR-FTIR) spectroscopy on a Bruker Tensor 27 instrument. Transmittance spectra were normalized by min-max scaling.

^{13}C Solid state nuclear magnetic resonance

Solid state ^{13}C cross polarization (CP) magic angle spinning (MAS) nuclear magnetic resonance (NMR) spectra were acquired on a Bruker AVIII 500 MHz spectrometer using a 4 mm MAS probe, spinning at 12.5 kHz. 4000 scans were accumulated for each spectrum.

Thermogravimetric analysis

Thermogravimetric analysis was performed on a Mettler Toledo TGA/DSC 3+ instrument under air atmosphere with a heating rate of 10 °C min^{-1} in a temperature range of 25-800 °C.

Density

The macroscopic density of COF films was determined by drying the samples in a 70 °C oven before weighing them, measuring their areas from an optical image, and their thicknesses by averaging micrometer gauge measurements at 10 points over the film. Due to the poor accuracy of the mean thickness determination, the macroscopic density should be regarded as a rough estimate. Skeletal density was measured by helium pycnometry on a Micromeritics AccuPycII 1340.

Swelling rate

Their swelling rates (mass increase compared to dry mass) were determined under two conditions. Firstly, after storage overnight in a water-filled desiccator, and secondly, after being wetted and gently pressed between filter papers to remove excess water.

Dye nanofiltration

The COF membranes were sandwiched between filter papers soaked in ethanol and then cut into circular filters using a Flux Beambox laser cutter. The filters were stored submerged in water until used. The filtration setup was custom-built from 3D-printed and off-the-shelf components (Figure S10). The COF filter was placed in the holder on a filter paper support. The effective area of the filter was 4.23 cm^2. A magnetic stirring bar was used to stir the feed side of the filter. It was fed with 10 ppm Congo red solution from a tank pressurized with N_2 at 3 bar. Retentate flow was restricted to 1-2 mL min^{-1} by a homemade valve. Permeate was collected in a series of 4 mL cuvettes. For each fraction, flux was determined by weight and collection time and concentration determined by UV-Vis spectroscopy on an Agilent Cary 60 instrument measuring absorbance at 500 nm (Figure S11). Filtration experiments were typically run for 1 hour and then stopped when the final cuvette was filled. The permeance and rejection values of the final cuvette were reported for each filter.

The Congo red adsorption capacity of a TpBd-C3 membrane was determined by placing 20 mg of dry membrane in 150 mL of 10 ppm Congo red solution and stirring for 8 hours. Three 1 mL portions of the solution was taken at during the period and the quantity of adsorbed dye determined by UV-Vis spectroscopy. The adsorption capacity of the membrane was determined to be ∼21 mg g^{-1} (Figure S12). The dry weight of the accessible membrane volume in the filtration experiment is ∼1 mg. As such the volume of 10 ppm Congo red solution containing enough dye to saturate the membrane is estimated to ∼2 ml.

H_2/CO_2 separation

Single gas permeance was determined using a custom-built constant volume variable pressure permeation system. A membrane with an effective area of 0.307 cm^2 was mounted and the system evacuated to below 10 Pa. The target gas (H_2 or CO_2) was applied on the feed side of the membrane at 0.3 MPa pressure relative to the vacuum applied to the permeate side. The pump valve was closed, and a high-precision pressure transducer was used to monitor the pressure rise on the permeate side. The permeance was calculated by equation 1.

$$P = \frac{V \cdot 273.15}{A \cdot T \cdot \Delta P \cdot 76} \cdot \frac{dp}{dt} \tag{1}$$

Where:

P is the gas permeance [1 GPU = 10^{-10}cm^3(STP)/(cm^2· s · cmHg)]

V is the feed side volume [cm^3]

A is the effective membrane area [cm^3]

T is the operating temperature [K]

ΔP is the transmembrane pressure difference [cmHg]

dp/dt is the rate of permeate side pressure rise [cmHg s^{-1}]

The ideal H_2/CO_2 selectivity was calculated by equation 2.

$$S_{H_2/CO_2} = \frac{P_{H_2}}{P_{CO_2}} \tag{2}$$

Mixed-gas separation performance was evaluated using a custom-built Wicke-Kallenbach setup. The feed side was pressurized at 0.2 MPa with a 1:1 volume ratio mixture of H_2 and CO_2. N_2 was used as a sweep gas at a flow rate of 5 mL min^{-1}. The permeate stream was analyzed in real time using an online gas chromatograph equipped with a thermal conductivity detector. The relative concentrations of H_2 and CO_2 in the permeate stream were determined. Based on the known membrane area, thickness, operating temperature, and pressures, the permeability and selectivity of the membrane were calculated using equations

3-4.

$$P = \frac{273.15 \cdot V_p \cdot Y_A}{A \cdot T(p_x \cdot x_A \cdot \varphi_{x_A} - p_y \cdot y_A \cdot \varphi_{y_A})} \tag{3}$$

$$\alpha_{A/B} = \frac{y_A}{y_B} \cdot \frac{x_B}{x_A} \tag{4}$$

Where:

P is the gas permeance [1 GPU = 10^{-10}cm^3(STP)/(cm^2$\cdot$ s $\cdot$ cmHg)]

A is the effective membrane area [cm^3]

V_p is volumetric flow rate of the permeated gas [mL s^{-1}]

T is the operating temperature [K]

p_x and p_y are the feed and permeate side pressures [cmHg]

x_A and y_A are the mole fractions of component A on the feed and permeate sides

φ is the fugacity coefficient

α is the separation factor

Supplementary data

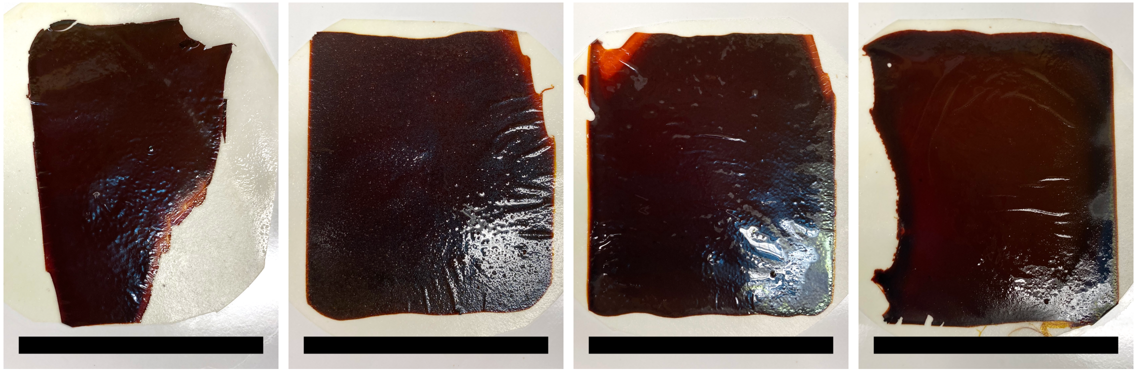

(a) TpBd-DCM (b) TpBd-IPA (c) TpBd-THF (d) TpBd-water

Figure S1: Optical images of TpBd films synthesized from suspensions in different solvents. (Scale bar: 10 cm)

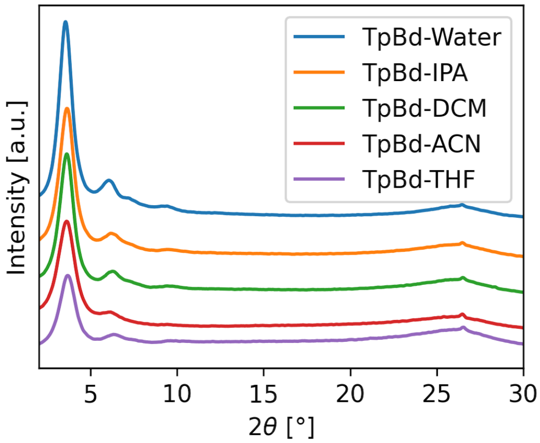

Figure S2: PXRD patterns of TpBd films synthesized from suspensions in different solvents.

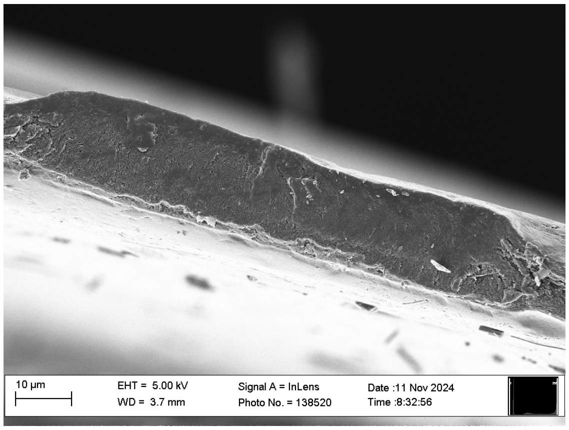

(a) ACN

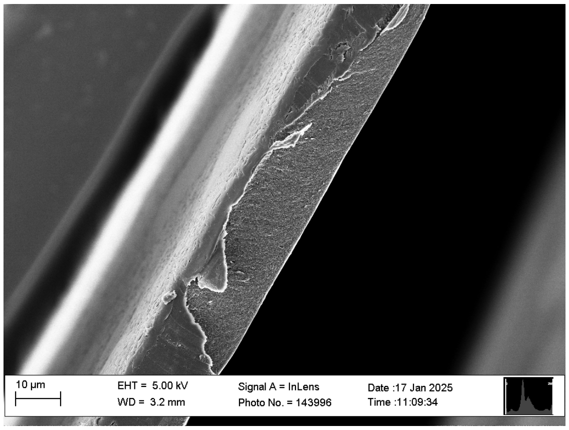

(b) DCM

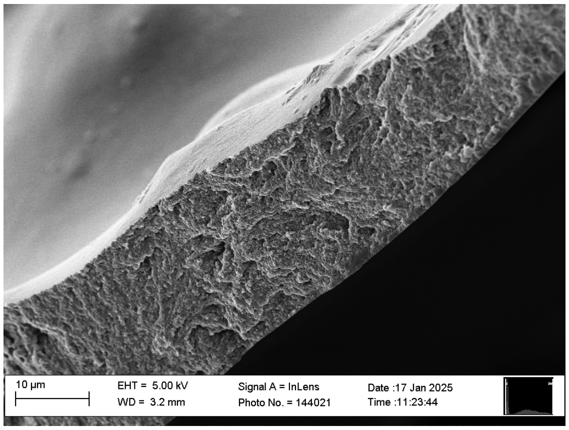

(c) IPA

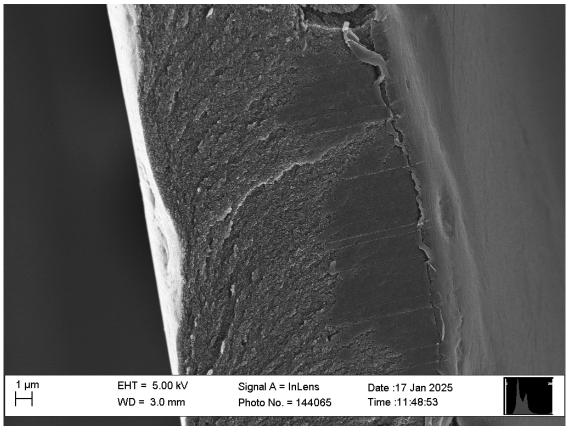

(d) THF

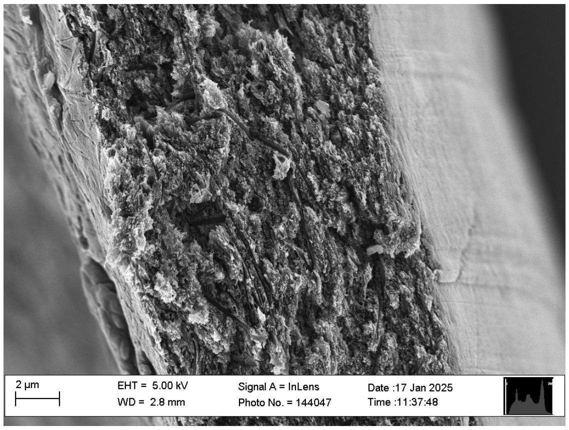

(e) Water

Figure S3: SEM cross section of TpBd films synthesized from suspensions in different solvents.

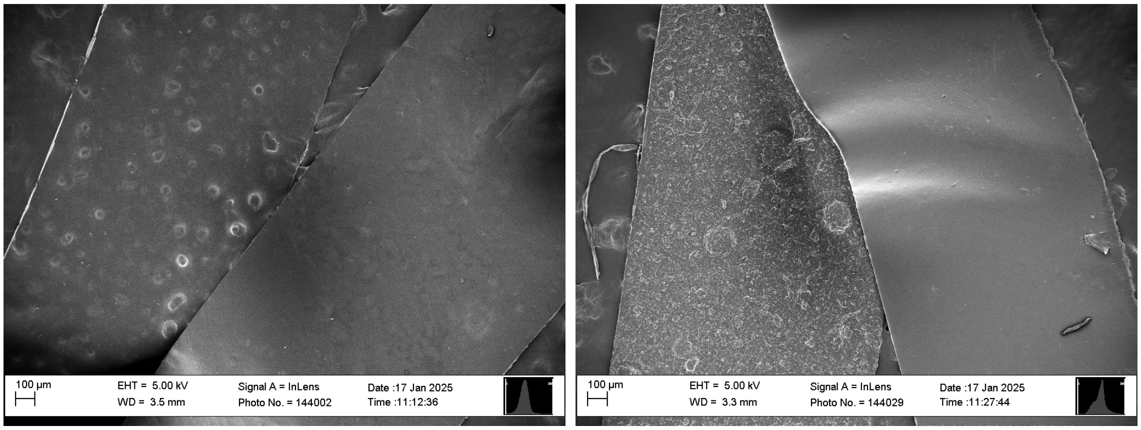

(a) TpBd-IPA (b) TpBd-water

Figure S4: SEM top view images of TpBd-IPA and TpBd-water. The top face is on the left side and the bottom face is on the right side in the respective image. The top faces of these films are uneven, unlike for the other solvents we used. In the case of TpBd-IPA it is a result of the Bd not being fully dispersed. In the case of TpBd-water, it is likely a result of the presence of stable intermediate fibers as described for TpBd-C4 in the main manuscript.

Table S1: Pawley refinement parameters of COFs. Source of structure data is referenced for each COF.

	**TpPa**[S1]	**TpBd**[S2]	**TpTd**[S2]	**TpAzo**[S3]
**Crystal system**	Hexagonal	Hexagonal	Hexagonal	Hexagonal
**Space group**	6/m	6/m	6/m	6/m
a [Å]	21.70	29.63	37.90	32.25
b [Å]	21.70	29.63	37.90	32.25
c [Å]	3.30	3.27	3.52	3.28
α [°]	90	90	90	90
β [°]	90	90	90	90
γ [°]	120	120	120	120
R_p	2.46	1.40	1.71	3.05
R_{wp}	3.96	2.06	3.07	4.46
GoF	0.22	0.13	0.19	0.27

	**TpBd(NO$_2$)$_2$**[S4]	**TpAq**[S5]	**TpPa(SO$_3$)**[S6]	**TpTta**[S4,S7]
**Crystal system**	Hexagonal	Hexagonal	Hexagonal	Hexagonal
**Space group**	P6	6/m	6/m	6/m
a [Å]	28.03	28.76	23.60	18.27
b [Å]	28.03	28.76	23.60	18.27
c [Å]	3.60	3.64	5.46	3.47
α [°]	90	90	90	90
β [°]	90	90	90	90
γ [°]	120	120	120	120
R_p	3.01	4.94	1.79	1.76
R_{wp}	4.43	7.07	2.56	2.36
GoF	0.23	0.36	0.12	0.13

Table S2: BET surface areas of our COF films and examples of the highest previously reported for COF powders and films.

Material	S_{BET} [m^2 g^{-1}]	Sample form	Synthesis method	Reference
TpBd-540 nm	1523	Film	Precursor casting	This work
TpBd-3.5 µm	1472	Film	Precursor casting	This work
TpBd-18 µm	1014	Film	Precursor casting	This work
TpBd	2157	Powder	Solvothermal	S8
TpBd	1883	Powder	Solvothermal	S9
TpBd	1850	Powder	Solvothermal	S10
TpBd	1536	Powder	Monomer exchange	S11
TpBd	1435	Powder	Solvothermal	S12
TpBd	1400	Powder	Baking method	S13
TpBd	800	Film	Baking method	S14
TpPa	1529	Film	Precursor casting	This work
TpPa	1432	Powder	Baking method	S13
TpPa	1163	Powder	Emulsion polymerization	S15
TpPa	1117	Powder	Solvothermal	S12
TpPa	1105	Powder	Room temperature aminocatalytic	S16
TpPa	742	Film	Vapor assisted interfacial	S17
TpPa	610	Film	Liquid interfacial	S18
TpTd	1433	Film	Precursor casting	This work
TpTd	1266	Powder	Solvothermal	S8
TpTd	1020	Powder	Baking method	S13
TpTd	971	Film	Baking method	S14
TpAzo	2226	Film	Precursor casting	This work
TpAzo	3038	Powder	Baking method	S13
TpAzo	2093	Film	Residual crystallization	S19
TpAzo	2033	Film	Baking method	S20
TpAzo	1855	Powder	Solvothermal	S21
TpAzo	1520	Powder	Solvothermal	S8
TpAzo	1400	Film	Baking method	S14
TpBd(NO_2)$_2$	1455	Film	Precursor casting	This work
TpBd(NO_2)$_2$	769	Powder	Baking method	S13
TpBd(NO_2)$_2$	608	Powder	Solvothermal	S22
TpBd(NO_2)$_2$	298	Powder	Solvothermal	S23
TpBd(NO_2)$_2$	295	Powder	Solvothermal	S24
TpBd(NO_2)$_2$	287	Powder	Solvothermal	S25

(Continued on next page)

Table S2 (Continued): BET surface areas of our COF films and examples of the highest previously reported for COF powders and films.

Material	S_{BET} [m^2 g^{-1}]	Sample form	Synthesis method	Reference
TpAq	1914	Film	Precursor casting	This work
TpAq	1800	Powder	Solvothermal	S26
TpAq	1180	Powder	Solvothermal	S27
TpAq	1140	Powder	Solvothermal	S28
TpAq	1027	Powder	Baking method	S13
TpAq	930	Film	Baking method	S14
TpAq	824	Powder	Solvothermal	S29
$TpPa(SO_3)$	498	Film	Precursor casting	This work
$TpPa(SO_3)$	454	Film	Vapor assisted interfacial	S30
$TpPa(SO_3)$	351	Powder	Solvothermal	S31
$TpPa(SO_3)$	251	Powder	Solvothermal	S32
$TpPa(SO_3)$	215	Powder	Solvothermal	S33
$TpPa(SO_3)$	159	Powder	Solvothermal	S34
$TpPa(SO_3)$	116	Powder	Baking method	S35
$TpPa(SO_3)$	57	Film	Precursor casting	S36
TpTta	779	Film	Precursor casting	This work
TpTta	1262	Powder	Solvothermal	S37
TpTta	1205	Powder	Solvothermal	S38
TpTta	1148	Powder	Monomer exchange	S11
TpTta	1323	Powder	Solvothermal	S39
TpTta	1037	Powder	Solvothermal	S40
TpTta	1014	Powder	Solvothermal	S41
TpTta	1012	Powder	Solvothermal	S42
TpTta	962	Powder	Solvothermal	S43
TpTta	907	Powder	Solvothermal	S44
TpTta	905	Powder	Solvothermal	S45
TpTta	896	Powder	Solvothermal	S46
TpTta	879	Powder	Solvothermal	S47
TpTta	867	Powder	Solvothermal	S8
TpTta	825	Powder	Baking method	S13
TpTta	448	Film	Baking method	S20
TpTta	298	Film	Liquid interfacial	S48

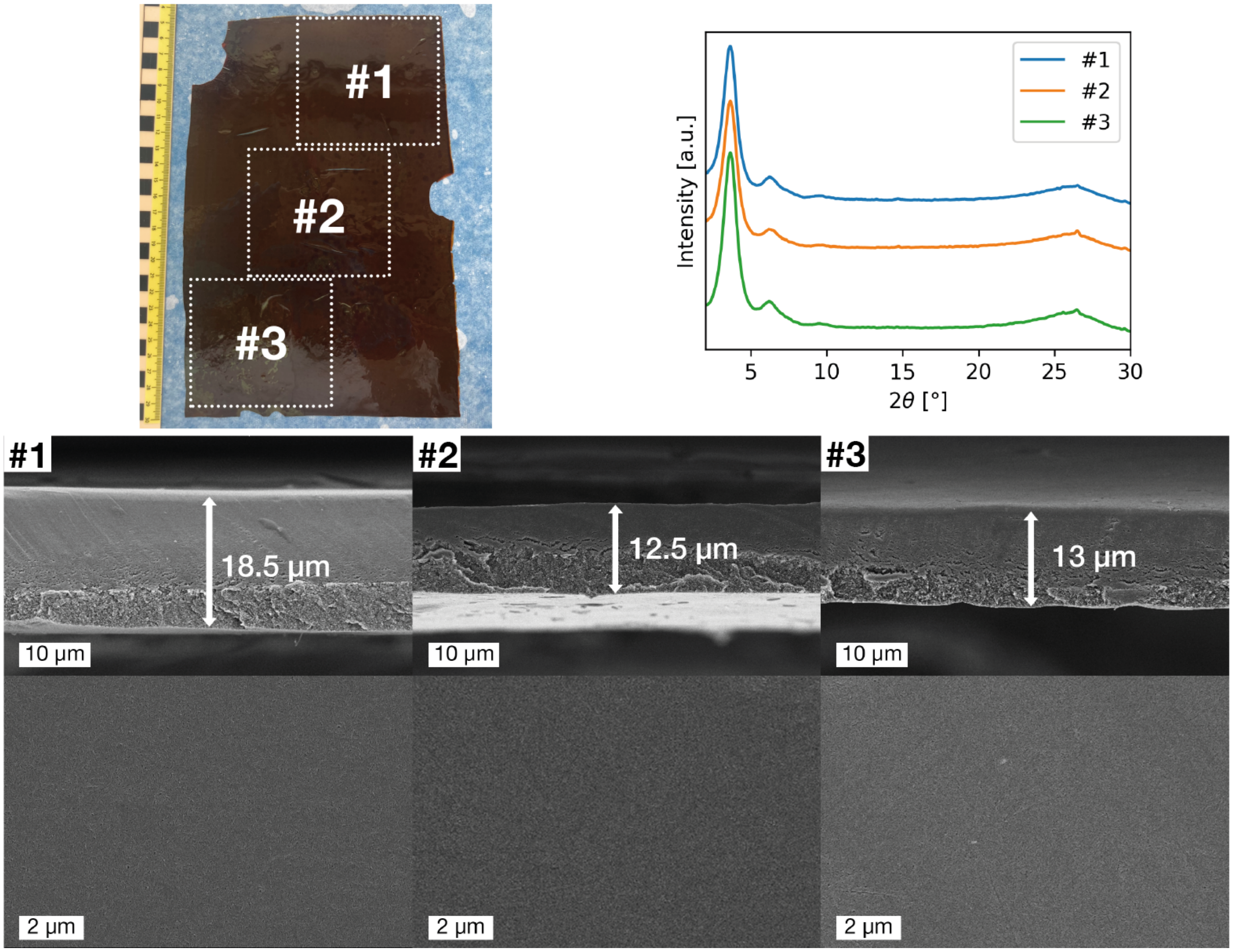

Figure S5: Optical image of large TpBd COF film with sections (10 x 8 cm large) #1, #2, and #3 marked. XRD patterns of material from each section. SEM cross sections and top view images of material from the center of each region.

Table S3: Mechanical properties of our films determined by tensile testing. TpBd-C1 was tested with 3 replicates. TpBd-C2, TpBd-C3 and TpBd-C4 were tested with 5 replicates each.

Sample	Young's modulus [GPa]	Ultimate strength [MPa]	Elongation at break [%]
TpBd-C1	1.88 ± 0.21	43.42 ± 17.49	2.87 ± 1.38
TpBd-C2	1.25 ± 0.17	47.00 ± 14.36	4.97 ± 1.72
TpBd-C3	1.71 ± 0.25	59.69 ± 15.46	4.39 ± 1.29
TpBd-C4	0.55 ± 0.07	10.86 ± 4.50	2.43 ± 1.15

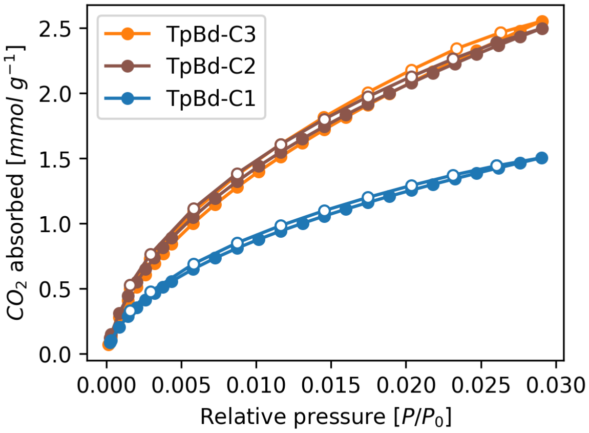

Figure S6: CO_2 sorption isotherms recorded at 0 °C. The Langmuir surface areas determined from the adsorption isotherms of TpBd-C1, -C2, and -C3 are 144, 248, and 254 m^2 g^{-1} respectively.

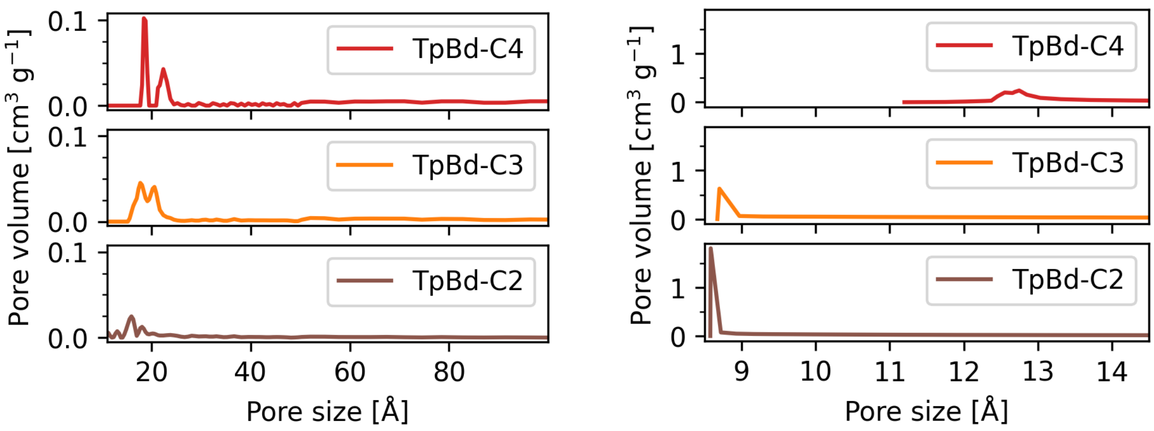

(a) DFT pore size distributions

(b) HK pore size distributions

Figure S7: DFT and HK pore size distributions calculated from N_2 adsorption isotherms.

Table S4: Reported mechanical properties of various free-standing COF films, determined by tensile testing.

Material	Young's modulus [GPa]	Ultimate strength [MPa]	Synthesis method
TfBd[S49]	4.5	220	Interfacial polymerization
TpPa(SO_3)[S50]	0.456	91.2	Nanosheet assembly
COF-QA-6[S51]	0.757	53	Interfacial polymerization
TpPa(SO_3)[S52]	0.606	24	Nanosheet assembly
TpBd$(SO_3)_2$[S53]	0.670	20	Interfacial polymerization
TpDa[S54]	0.147	12.5	Baking method
NKCOF-47[S55]	0.125	3.84	Interfacial polymerization

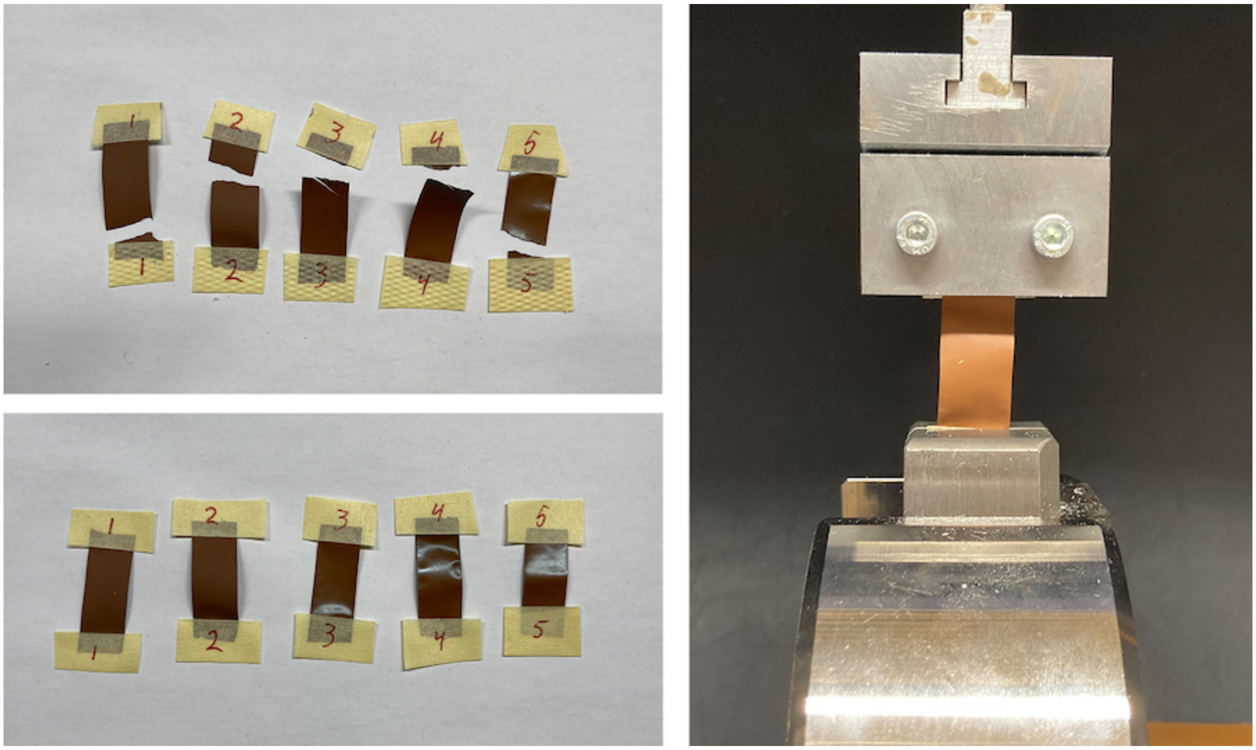

Figure S8: TpBd samples before, during and after tensile testing.

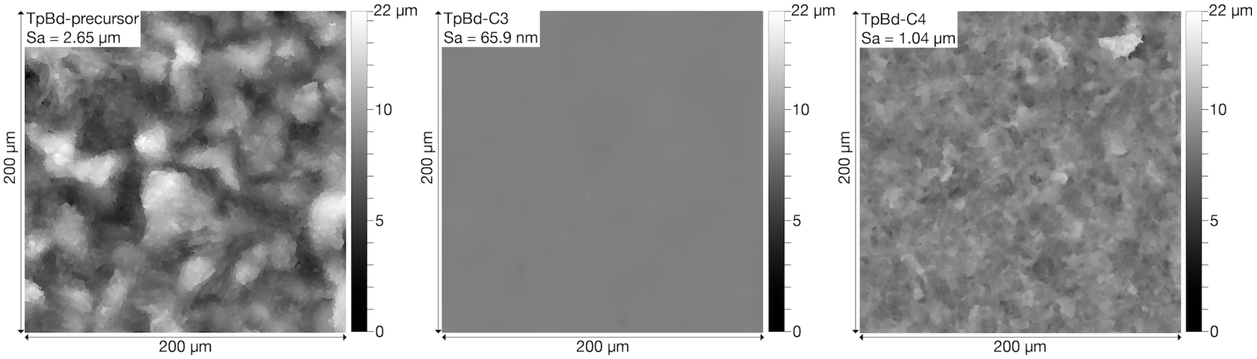

Figure S9: Coherence scanning interferometry maps of film top surfaces. (Sa - Arithmetical Mean Height)

Table S5: Densities and swelling rates of COF films.

	TpBd-C3	TpBd-C4
Macroscopic density [g cm^{-3}]	0.84	0.85
Skeletal density [g cm^{-3}]	1.29	1.39
Swelling rate (humid storage)	38%	54%
Swelling rate (wetted)	350%	1036%

Table S6: Nanofiltration performance filtering Congo Red (CR) from aqueous solution. Each membrane was tested with 3 replicates.

Sample	Water permeance [L $m^{-2}h^{-1}bar^{-1}$]	CR rejection [%]
TpBd-C1	4.3 ± 0.56	99.2 ± 0.7
TpBd-C2	15.4 ± 1.7	99.7 ± 0.4
TpBd-C3	29.9 ± 2.8	99.0 ± 1.0
TpBd-C4	86.4 ± 8.8	90.4 ± 5.6

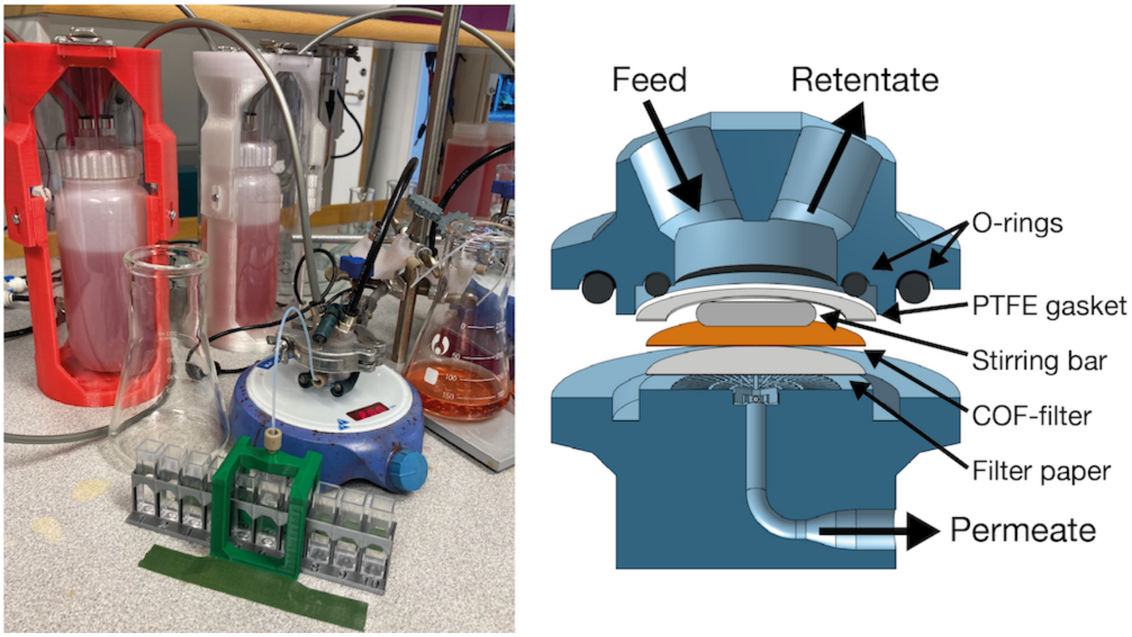

Figure S10: Optical image of filtration setup during operation and schematic cross section of filter holder.

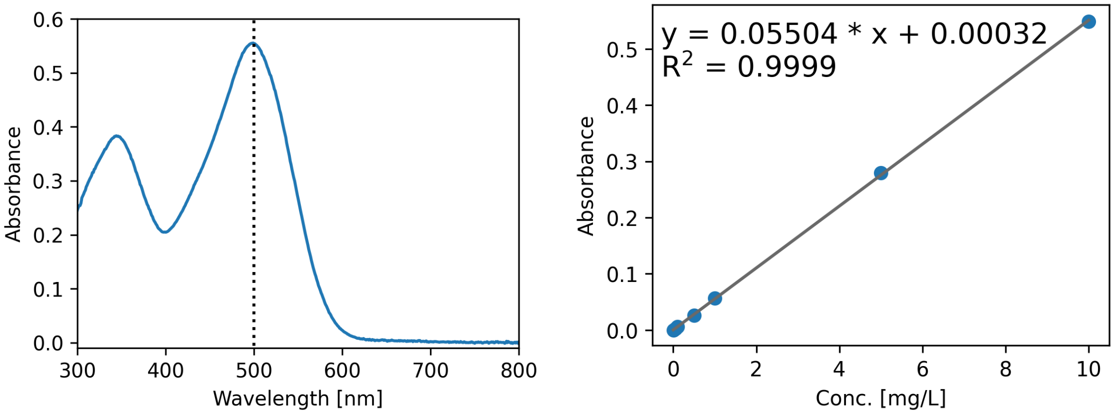

Figure S11: UV-vis. spectrum of 10 mg L^{-1} Congo red solution and the standard curve used for concentration determination in the Congo red nanofiltration experiments.

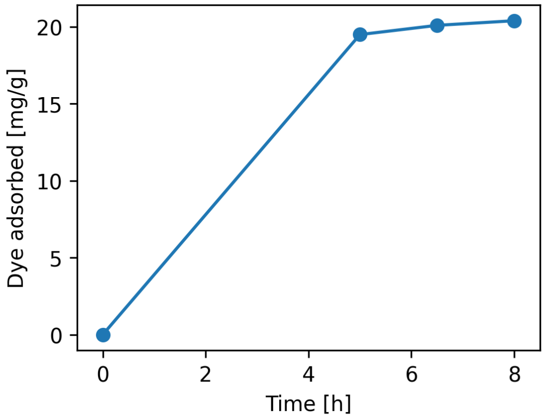

Figure S12: Adsorption of Congo red dye from solution in TpBd-C3 film.

Table S7: Reported mixed gas H_2/CO_2 separation performance of various pristine COF, COF composite, and vertically aligned COF membranes.

Material	SF	$P(H_2)$ [GPU]	Membrane type	Synthesis method	Reference
Pristine COF membranes					
TpBd-C2	11.0	2857	Free-standing	Precursor casting	This work
TAPB-PDA	3.8†	191	Free-standing	Powder pressing	S56
TpPa-Me	12.7	727	Free-standing	Liquid interfacial	S57
COF-300	13.8	4319	Free-standing	Vapor assisted	S58
N-COF	11.0	5160	Free-standing	Vapor assisted	S58
DMTA-COF	6.0	2688	Substrate-bound	In situ growth	S59
DMTA-COF	8.3	1857	Substrate-bound	In situ growth	S60
LZU1	6.0	3734	Substrate-bound	In situ growth	S61
ACOF-1	14.1	2046	Substrate-bound	In situ growth	S61
COF composite membranes					
LZU1/ACOF-1	24.2	669.1	COF/COF composite	Bilayer in situ growth	S61
TpEBr/TpPa(SO_3)	22.6	2566	COF/COF composite	Nanosheet assembly	S62
TpTg/TpPa(SO_3)	26.0	2163	COF/COF composite	Multilayer interfacial	S63
ZIF-67/TpPa	34.9	3671	COF/MOF composite	MOF-in-COF in-situ-growth	S64
ZIF-67/TpBd	27.9	3773	COF/MOF composite	MOF-in-COF in-situ-growth	S64
COF-300/Zn-DMOF	12.6	1340	COF/MOF composite	Bilayer in situ growth	S59
TpPa/GO	25.6	3145	COF/GO composite	Nanosheet assembly	S65
Vertically aligned COF membranes					
COF-LZU1	31.6	3655	Vertically aligned	In situ growth	S66
TFB-BD	25.6	3802	Vertically aligned	In situ growth	S66

SF - H_2/CO_2 separation factor, $P(H_2)$ - H_2 permeance
† - ideal selectivity determined from single gas permeance

Table S8: TpBd-C2 gas permeation result for H_2/CO_2 (1:1, v/v) at 0.2 MPa transmembrane pressure with varying temperature.

T [°C]	$P(H_2)$ [GPU]	$P(CO_2)$ [GPU]	$S(H_2/CO_2)$
25	2857 ± 26	260 ± 5	11.0 ± 0.3
30	2668 ± 28	296 ± 2	9.0 ± 0.2
40	2549 ± 17	296 ± 2	8.6 ± 0.1
50	2346 ± 19	296 ± 11	7.9 ± 0.4
60	2211 ± 20	285 ± 7	7.8 ± 0.2
70	2147 ± 23	274 ± 5	7.8 ± 0.2
80	2038 ± 8	268 ± 1	7.6 ± 0.0

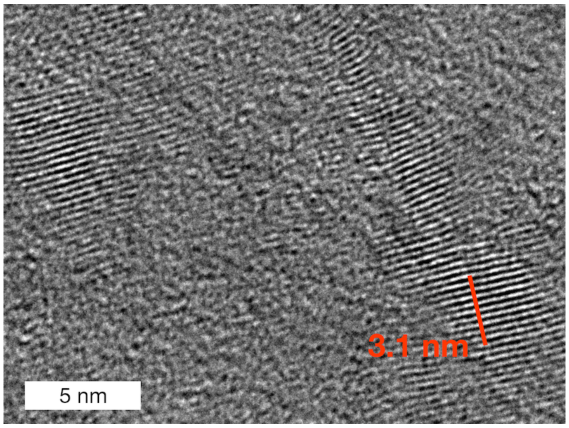

Figure S13: High-resolution TEM image of a TpBd-C2 film showing regions with parallel fringes. A distance of 3.1 nm, spanning 10 fringes, is annotated in the figure. The observed inter-fringe spacing of 3.1 Å is consistent with the expected inter-layer distance of TpBd-COF (∼3.3 Å).

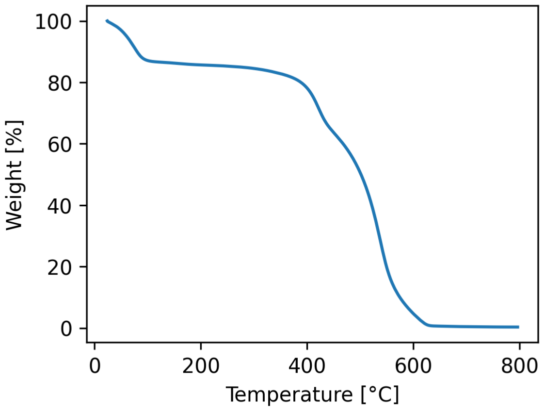

Figure S14: Thermogravimetric analysis of TpBd-C3 in air. Indicating stability up to ∼400°C.

References

(S1) Kandambeth, S.; Mallick, A.; Lukose, B.; Mane, M. V.; Heine, T.; Banerjee, R. Construction of Crystalline 2D Covalent Organic Frameworks with Remarkable Chemical (Acid/Base) Stability via a Combined Reversible and Irreversible Route. *J. Am. Chem. Soc.* **2012**, *134*, 19524–19527.

(S2) Biswal, B. P.; Chandra, S.; Kandambeth, S.; Lukose, B.; Heine, T.; Banerjee, R. Mechanochemical Synthesis of Chemically Stable Isoreticular Covalent Organic Frameworks. *J. Am. Chem. Soc.* **2013**, *135*, 5328–5331.

(S3) Chandra, S.; Kundu, T.; Kandambeth, S.; BabaRao, R.; Marathe, Y.; Kunjir, S. M.; Banerjee, R. Phosphoric Acid Loaded Azo (-N=N-) Based Covalent Organic Framework for Proton Conduction. *J. Am. Chem. Soc.* **2014**, *136*, 6570–6573.

(S4) Tong, M.; Lan, Y.; Yang, Q.; Zhong, C. Exploring the structure-property relationships of covalent organic frameworks for noble gas separations. *Chem. Eng. Sci.* **2017**, *168*, 456–464.

(S5) DeBlase, C. R.; Silberstein, K. E.; Truong, T.-T.; Abruña, H. D.; Dichtel, W. R. β-Ketoenamine-Linked Covalent Organic Frameworks Capable of Pseudocapacitive Energy Storage. *J. Am. Chem. Soc.* **2013**, *135*, 16821–16824.

(S6) Peng, Y.; Xu, G.; Hu, Z.; Cheng, Y.; Chi, C.; Yuan, D.; Cheng, H.; Zhao, D. Mechanoassisted Synthesis of Sulfonated Covalent Organic Frameworks with High Intrinsic Proton Conductivity. *ACS Appl. Mater. Interfaces.* **2016**, *8*, 18505–18512.

(S7) Gomes, R.; Bhaumik, A. A new triazine functionalized luminescent covalent organic framework for nitroaromatic sensing and CO 2 storage. *RSC adv.* **2016**, *6*, 28047–28054.

(S8) Wang, R.; Kong, W.; Zhou, T.; Wang, C.; Guo, J. Organobase modulated synthesis of high-quality β-ketoenamine-linked covalent organic frameworks. *Chem. Commun.* **2021**, *57*, 331–334.

(S9) Tan, J.; Namuangruk, S.; Kong, W.; Kungwan, N.; Guo, J.; Wang, C. Manipulation of Amorphous-to-Crystalline Transformation: Towards the Construction of Covalent Organic Framework Hybrid Microspheres with NIR Photothermal Conversion Ability. *Angew. Chem. Int. Ed.* **2016**, *128*, 14185–14190.

(S10) Kong, W.; Wan, J.; Namuangruk, S.; Guo, J.; Wang, C. Water-soluble metalated covalent organic nanobelts with improved bioavailability for protein transportation. *Sci. Rep.* **2018**, *8*.

(S11) Daugherty, M. C.; Vitaku, E.; Li, R. L.; Evans, A. M.; Chavez, A. D.; Dichtel, W. R. Improved synthesis of β-ketoenamine-linked covalent organic frameworks: Via monomer exchange reactions. *Chem. Commun.* **2019**, *55*, 2680–2683.

(S12) Kou, J.; Wang, G.; Guo, H.; Li, L.; Fang, J.; Ma, J.; Dong, Z. Photocatalytic benzylamine coupling dominated by modulation of linkers in donor-acceptor covalent organic frameworks. *Appl. Catal. B: Environ.* **2024**, *352*.

(S13) Karak, S.; Kandambeth, S.; Biswal, B. P.; Sasmal, H. S.; Kumar, S.; Pachfule, P.; Banerjee, R. Constructing ultraporous covalent organic frameworks in seconds via an organic terracotta process. *J. Am. Chem. Soc.* **2017**, *139*, 1856–1862.

(S14) Kandambeth, S.; Biswal, B. P.; Chaudhari, H. D.; Rout, K. C.; H., S. K.; Mitra, S.; Karak, S.; Das, A.; Mukherjee, R.; Kharul, U. K.; Banerjee, R. Selective Molecular Sieving in Self-Standing Porous Covalent-Organic-Framework Membranes. *Adv. Mater.* **2017**, *29*.

(S15) Zhang, J.; Cheng, C.; Guan, L.; Jiang, H. L.; Jin, S. Rapid Synthesis of Covalent Organic Frameworks with a Controlled Morphology: An Emulsion Polymerization

Approach via the Phase Transfer Catalysis Mechanism. *J. Am. Chem. Soc.* **2023**, *145*, 21974–21982.

(S16) Zhang, J.; Cheng, C.; Guan, L.; Jiang, H. L.; Jin, S. Rapid Synthesis of Covalent Organic Frameworks with a Controlled Morphology: An Emulsion Polymerization Approach via the Phase Transfer Catalysis Mechanism. *J. Am. Chem. Soc.* **2023**, *145*, 21974–21982.

(S17) Khan, N. A.; Zhang, R.; Wang, X.; Cao, L.; Azad, C. S.; Fan, C.; Yuan, J.; Long, M.; Wu, H.; Olson, M. A.; Jiang, Z. Assembling covalent organic framework membranes via phase switching for ultrafast molecular transport. *Nat. Commun.* **2022**, *13*.

(S18) Wu, Y.; Wang, Y.; Xu, F.; Qu, K.; Dai, L.; Cao, H.; Xia, Y.; Lei, L.; Huang, K.; Xu, Z. Solvent-induced interfacial polymerization enables highly crystalline covalent organic framework membranes. *J. Membr. Sci.* **2022**, *659*.

(S19) Mahato, A. K.; Bag, S.; Sasmal, H. S.; Dey, K.; Giri, I.; Linares-Moreau, M.; Carbonell, C.; Falcaro, P.; Gowd, E. B.; Vijayaraghavan, R. K.; Banerjee, R. Crystallizing Sub 10 nm Covalent Organic Framework Thin Films via Interfacial-Residual Concomitance. *J. Am. Chem. Soc.* **2021**, *143*, 20916–20926.

(S20) Dey, K.; H., S. K.; Chahande, A. M.; Banerjee, R. Nanoparticle Size-Fractionation through Self-Standing Porous Covalent Organic Framework Films. *Angew. Chem. Int. Ed.* **2020**, *132*, 1177–1181.

(S21) Xiong, K.; Wang, Y.; Huang, F.; Zhang, K.; Zeng, B.; Lang, X. Tailoring β-ketoenamine covalent organic framework with azo for blue light-driven selective oxidation of amines with oxygen. *J. Colloid Interface Sci.* **2024**, *665*, 252–262.

(S22) Jin, W.; Li, S.; Zhou, H.; Ma, S.; Zhu, L.; Kong, D. Pore Wall Engineered Polar COFs as Size-Matched Nanotraps Enable Highly Efficient Capture of Long-Chain Alkylamines. *Adv. Funct. Mater.* **2024**,

(S23) Zhong, X.; Ji, Z.; Ling, Q.; Sun, L.; Hu, B. Immobilization of laccase on different functional groups and degree of conjugation modified COFs for simultaneous photochemical-enzymatic removal of 2,4-dichlorophenol and uranium (U(vi)). *J. Mater. Chem. C* **2025**,

(S24) Chandra, S.; Kandambeth, S.; Biswal, B. P.; Lukose, B.; Kunjir, S. M.; Chaudhary, M.; Babarao, R.; Heine, T.; Banerjee, R. Chemically stable multilayered covalent organic nanosheets from covalent organic frameworks via mechanical delamination. *J. Am. Chem. Soc.* **2013**, *135*, 17853–17861.

(S25) Xiong, M.; Wang, B.; Wang, H.; Xu, F.; Zeng, Y.; Ren, H.; Zeng, H. Probing the adsorption behavior and mechanism of NO2 and NH2 functionalized covalent organic frameworks (COFs) for removal of bisphenol A. *Microporous Mesoporous Mater.* **2022**, *346*.

(S26) Deblase, C. R.; Silberstein, K. E.; Truong, T. T.; Abruña, H. D.; Dichtel, W. R. β-ketoenamine-linked covalent organic frameworks capable of pseudocapacitive energy storage. *J. Am. Chem. Soc.* **2013**, *135*, 16821–16824.

(S27) Mishra, B.; Biswal, S.; Ussama, M.; Haider, M. A.; Tripathi, B. P. Rationally designed oxygen vacant TiO2 decorated with covalent organic framework for enhanced electrocatalytic nitrogen reduction to ammonia. *Appl. Catal. B: Environ.* **2024**, *342*.

(S28) Vitaku, E.; Gannett, C. N.; Carpenter, K. L.; Shen, L.; Abruña, H. D.; Dichtel, W. R. Phenazine-Based Covalent Organic Framework Cathode Materials with High Energy and Power Densities. *J. Am. Chem. Soc.* **2020**, *142*, 16–20.

(S29) Cai, S.; Ma, R.; Ke, W.; Zhang, H.; Liu, Y.; Jiao, M.; Tian, Y.; Fang, Y.; Wu, M.; Zhou, Z. Flower-like covalent organic frameworks as host materials for high-performance lithium-sulfur batteries. *Chem. Eng. J.* **2024**, *491*.

(S30) Khan, N. A.; Luo, M.; Zha, X.; Azad, C. S.; Lu, J.; Chen, J.; Fan, C.; Rahman, A. U.; Olson, M. A.; Jiang, Z.; Wang, D. Water/Vapor Assisted Fabrication of Large-Area Superprotonic Conductive Covalent Organic Framework Membranes. *Small* **2023**, *19*.

(S31) Zhai, L.; Yao, Y.; Ma, B.; Hasan, M. M.; Han, Y.; Mi, L.; Nagao, Y.; Li, Z. Accumulation of Sulfonic Acid Groups Anchored in Covalent Organic Frameworks as an Intrinsic Proton-Conducting Electrolyte. *Macromol. Rapid Commun.* **2022**, *43*.

(S32) Mondal, T.; Seth, J.; Sarkar, S.; Islam, S. M. A SO3H-group anchored covalent organic framework for the synthesis of hydroxy carbamates in a single step utilizing CO2. *New J. Chem.* **2023**, *47*, 8885–8893.

(S33) Chandra, S.; Kundu, T.; Dey, K.; Addicoat, M.; Heine, T.; Banerjee, R. Interplaying Intrinsic and Extrinsic Proton Conductivities in Covalent Organic Frameworks. *Chem. Mater.* **2016**, *28*, 1489–1494.

(S34) Peng, Y.; Hu, Z.; Gao, Y.; Yuan, D.; Kang, Z.; Qian, Y.; Yan, N.; Zhao, D. Synthesis of a Sulfonated Two-Dimensional Covalent Organic Framework as an Efficient Solid Acid Catalyst for Biobased Chemical Conversion. *ChemSusChem* **2015**, *8*, 3208–3212.

(S35) Jin, W.; Liu, J.; Huang, N.; Wang, Z.; Zhang, Y.; Peng, Y.; Gong, C.; Ok, Y. S.; Xu, Z. Ultrafast Selective Enrichment of Ammonia Nitrogen from Water Using Sulfonated Covalent Organic Frameworks Bearing Single Cu Sites. *ACS EST Engg.* **2023**, *3*, 1511–1520.

(S36) Liu, X.; Wang, J.; Shang, Y.; Yavuz, C. T.; Khashab, N. M. Ionic Covalent Organic Framework-Based Membranes for Selective and Highly Permeable Molecular Sieving. *J. Am. Chem. Soc.* **2024**, *146*, 2313–2318.

(S37) Chowdhury, S.; Sharma, A.; Das, P. P.; Rathi, P.; Siril, P. F. Fine-tuning covalent organic frameworks for structure-activity correlation via adsorption and catalytic studies. *J. Colloid Interface Sci.* **2024**, *665*, 988–998.

(S38) Rathi, P.; Chowdhury, S.; Das, P. P.; Keshri, A. K.; Chaudhary, A.; Siril, P. F. Pore-interface engineering improves doxorubicin loading to triazine-based covalent organic framework. *Mater. Adv.* **2023**, *5*, 136–142.

(S39) Gan, S. X.; Jia, C.; Qi, Q. Y.; Zhao, X. A facile and scalable synthetic method for covalent organic nanosheets: Ultrasonic polycondensation and photocatalytic degradation of organic pollutants. *Chem. Sci.* **2022**, *13*, 1009–1015.

(S40) Lin, X.; He, Y.; Zhang, Y.; Yu, W.; Lian, T. Sulfonated covalent organic frameworks (COFs) incorporated cellulose triacetate/cellulose acetate (CTA/CA)-based mixed matrix membranes for forward osmosis. *J. Membr. Sci.* **2021**, *638*.

(S41) Das, S. K.; Chandra, B. K.; Molla, R. A.; Sengupta, M.; Islam, S. M.; Majee, A.; Bhaumik, A. CuO grafted triazine functionalized covalent organic framework as an efficient catalyst for C-C homo coupling reaction. *Mol. Catal.* **2020**, *480*.

(S42) Hussain, M.; Saddique, A.; Devarayapalli, K. C.; Kim, B.; Cheong, I. W.; Lee, D. S. Constructing bifunctional and robust covalent organic frameworks via three-component one-pot Doebner reaction for Cr(VI) removal. *Appl. Catal. B: Environ.* **2024**, *344*.

(S43) Zhang, J.; Cao, Y.; Liu, W.; Cao, T.; Qian, J.; Wang, J.; Yao, X.; Iqbal, A.; Qin, W. Structural Engineering of Covalent Organic Frameworks Comprising Two Electron Acceptors Improves Photocatalytic Performance. *ChemSusChem* **2022**, *15*.

(S44) Xu, X.; Dong, S.; Lv, J.; Huang, G.; Chen, Q.; Bi, J. Interfacial C-N-S bridged SnS2/COF S-scheme heterojunction with upgraded near-infrared photo-activity for H2O2 synthesis. *Appl. Surf. Sci.* **2025**, *689*.

(S45) Miao, X.; Zhang, F.; Wang, Y.; Dong, X.; Lang, X. 2D β-ketoenamine-linked triazine covalent organic framework photocatalysis for selective oxidation of sulfides. *Sustain. Energy Fuels* **2023**, *7*, 1963–1973.

(S46) Liu, X.; Wang, M.; Ren, Y.; Liu, J.; Dai, H.; Yang, Q. Construction of modularized catalytic system for transfer hydrogenation: Promotion effect of hydrogen bonds. *Chin. J. Catal.* **2023**, *52*, 207–216.

(S47) Zhao, J.; Cao, Q.; Zhao, L.; Li, F.; yangyang xin; Liu, Y.; Zhang, W.; shi, X.; Wu, M.; Zhao, Y.; Fa, Y.; Liu, H. Optimisation of extraction performance for uranium with covalent organic frameworks of β-ketoenamines with different functional groups. *Chem. Eng. J.* **2024**, *500*.

(S48) Liang, L.; Su, L.; Zhang, X.; Wang, Y.; Ren, W.; Gao, X.; Zheng, L.; Lu, F. Synergistically regulating Zn-ion flux and accelerating ion transport kinetics via zincophilic covalent organic framework interlayer for stable Zn metal anode. *Chem. Eng. J.* **2024**, *485*.

(S49) Cheng, G.; Sui, C.; Hao, W.; Li, J.; Zhao, Y.; Miao, L.; Zhao, G.; Li, J.; Sang, Y.; Zhao, C.; Wen, L.; He, X.; Wang, C. Ultra-Strong Janus Covalent Organic Framework Membrane with Smart Response to Organic Vapor. *Small* **2024**, *20*, e2401635–n/a.

(S50) Cao, L.; Wu, H.; Cao, Y.; Fan, C.; Zhao, R.; He, X.; Yang, P.; Shi, B.; You, X.; Jiang, Z. Weakly Humidity-Dependent Proton-Conducting COF Membranes. *Adv. Mater.* **2020**, *32*, e2005565–n/a.

(S51) He, X.; Yang, Y.; Wu, H.; He, G.; Xu, Z.; Kong, Y.; Cao, L.; Shi, B.; Zhang, Z.; Tongsh, C.; Jiao, K.; Zhu, K.; Jiang, Z. De Novo Design of Covalent Organic Framework Membranes toward Ultrafast Anion Transport. *Adv. Mater.* **2020**, *32*, e2001284–n/a.

(S52) Huang, T.; Jiang, H.; Douglin, J. C.; Chen, Y.; Yin, S.; Zhang, J.; Deng, X.; Wu, H.; Yin, Y.; Dekel, D. R.; Guiver, M. D.; Jiang, Z. Single Solution-Phase Synthesis of Charged Covalent Organic Framework Nanosheets with High Volume Yield. *Angew. Chem. Int. Ed.* **2023**, *62*.

(S53) Wang, X.; Shi, B.; Yang, H.; Guan, J.; Liang, X.; Fan, C.; You, X.; Wang, Y.; Zhang, Z.; Wu, H.; Cheng, T.; Zhang, R.; Jiang, Z. Assembling covalent organic framework membranes with superior ion exchange capacity. *Nat. Commun.* **2022**, *13*, 1020–1020.

(S54) Khayum M, A.; Vijayakumar, V.; Karak, S.; Kandambeth, S.; Bhadra, M.; Suresh, K.; Acharambath, N.; Kurungot, S.; Banerjee, R. Convergent Covalent Organic Framework Thin Sheets as Flexible Supercapacitor Electrodes. *ACS Appl. Mater. Interfaces.* **2018**, *10*, 28139–28146.

(S55) Jia, S.; Hao, L.; Liu, Y.; Lin, E.; Liu, W.; Yang, Y.; Tian, Y.; Peng, Y.; Cheng, P.; Chen, Y.; Zhang, Z. Freestanding Hydrophilic/Hydrophobic Janus Covalent Organic Framework Membranes for Highly Efficient Solar Steam Generation. *ACS Materials Lett.* **2023**, *5*, 458–465.

(S56) Martín-Illán, J. A.; Suárez, J. A.; Gómez-Herrero, J.; Ares, P.; Gallego-Fuente, D.; Cheng, Y.; Zhao, D.; Maspoch, D.; Zamora, F. Ultralarge Free-Standing Imine-Based Covalent Organic Framework Membranes Fabricated via Compression. *Adv. Sci.* **2022**, *9*, e2104643–n/a.

(S57) Zheng, W.; Hou, J.; Liu, C.; Liu, P.; Li, L.; Chen, L.; Tang, Z. Melamine-Doped Covalent Organic Framework Membranes for Enhanced Hydrogen Purification. *Chem. Asian J.* **2021**, *16*, 3624–3629.

(S58) Li, B.; Wang, Z.; Gao, Z.; Suo, J.; Xue, M.; Yan, Y.; Valtchev, V.; Qiu, S.; Fang, Q. Self-Standing Covalent Organic Framework Membranes for H2/CO2 Separation. *Adv. Funct. Mater.* **2023**, *33*.

(S59) Fu, J.; Das, S.; Xing, G.; Ben, T.; Valtchev, V.; Qiu, S. Fabrication of COF-MOF Composite Membranes and Their Highly Selective Separation of H2/CO2. *J. Am. Chem. Soc.* **2016**, *138*, 7673–7680.

(S60) Fu, J.; Ben, T. Fabrication of a Novel Covalent Organic Framework Membrane and Its Gas Separation Performance. *Acta Chim. Sin.* **2020**, *78*, 805–814.

(S61) Fan, H.; Mundstock, A.; Feldhoff, A.; Knebel, A.; Gu, J.; Meng, H.; Caro, J. Covalent Organic Framework-Covalent Organic Framework Bilayer Membranes for Highly Selective Gas Separation. *J. Am. Chem. Soc.* **2018**, *140*, 10094–10098.

(S62) Ying, Y.; Tong, M.; Ning, S.; Ravi, S. K.; Peh, S. B.; Tan, S. C.; Pennycook, S. J.; Zhao, D. Ultrathin Two-Dimensional Membranes Assembled by Ionic Covalent Organic Nanosheets with Reduced Apertures for Gas Separation. *J. Am. Chem. Soc.* **2020**, *142*, 4472–4480.

(S63) Ying, Y.; Peh, S. B.; Yang, H.; Yang, Z.; Zhao, D. Ultrathin Covalent Organic Framework Membranes via a Multi-Interfacial Engineering Strategy for Gas Separation. *Adv. Mater.* **2022**, *34*, e2104946–n/a.

(S64) Fan, H.; Peng, M.; Strauss, I.; Mundstock, A.; Meng, H.; Caro, J. MOF-in-COF molecular sieving membrane for selective hydrogen separation. *Nat. Commun.* **2021**, *12*, 38–10.

(S65) Tang, Y.; Feng, S.; Fan, L.; Pang, J.; Fan, W.; Kong, G.; Kang, Z.; Sun, D. Covalent organic frameworks combined with graphene oxide to fabricate membranes for H2/CO2 separation. *Sep. Purif. Technol.* **2019**, *223*, 10–16.

(S66) Fan, H.; Peng, M.; Strauss, I.; Mundstock, A.; Meng, H.; Caro, J. High-Flux Vertically Aligned 2D Covalent Organic Framework Membrane with Enhanced Hydrogen Separation. *J. Am. Chem. Soc.* **2020**, *142*, 6872–6877.
